# Therapeutic Oligonucleotides for Neurodegenerative Diseases: Aptamer Strategies and Clay Nanoparticle‐Based Delivery

**DOI:** 10.1002/tcr.202500126

**Published:** 2025-10-06

**Authors:** Valentina Arciuolo, Federica D’Aria, Maria Rita Caruso, Martina Maria Calvino, Jussara Amato, Giuseppe Lazzara, Stefana Milioto, Concetta Giancola, Giuseppe Cavallaro, Bruno Pagano

**Affiliations:** ^1^ Department of Pharmacy University of Naples Federico II 80131 Naples Italy; ^2^ Department of Physics and Chemistry ‐ Emilio Segrè University of Palermo 90128 Palermo Italy

**Keywords:** aptamers, drug delivery, G‐quadruplexes, nanomaterials, nucleic acids

## Abstract

Aptamers have emerged as promising therapeutic oligonucleotides (TOs) due to their structural adaptability, high binding affinity, and remarkable specificity toward diverse biological targets. Among them, G‐quadruplex‐forming aptamers stand out for their unique secondary structures and distinct chemical properties. Their potential in neurodegenerative diseases such as Alzheimer's, Parkinson's, and Huntington's lies in their ability to inhibit protein aggregation and modulate pathogenic pathways. However, their application is hindered by enzymatic degradation and limited membrane permeability. To overcome these issues, chemical modifications, such as backbone and sugar alterations, and nanomaterial‐based delivery strategies have been developed. Notably, clay nanoparticles, such as halloysite nanotubes and montmorillonite, have gained attention as effective carriers for TOs, enhancing their structural stability and bioavailability. This review discusses recent advancements in aptamer‐based TOs, with a focus on G‐quadruplex‐forming oligonucleotides, their therapeutic potential in neurodegenerative diseases, and innovative nanocarrier systems that can improve their stability and targeted delivery. Finally, it highlights current challenges and future directions in the chemical design and formulation of aptamer‐based therapeutics for targeted applications.

## Graphical abstract

This review explores aptamers as therapeutic oligonucleotides targeting neurodegenerative diseases, including Alzheimer's, Parkinson's, and Huntington's, with a focus on G‐quadruplex‐forming sequences. It discusses chemical modifications that enhance aptamer stability and function, alongside the use of clay nanoparticles, such as halloysite nanotubes and montmorillonite, as nanocarriers for improved delivery. These strategies represent a promising synergy between nucleic acid chemistry and nanotechnology.

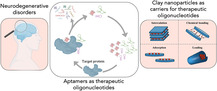

## 1 Introduction

Nucleic acid aptamers are highly promising tools in biomedical research and therapeutic applications due to their exceptional specificity and affinity toward a wide range of targets, ranging from small molecules to complex biological structures.^[^ ^1^ ^]^ Aptamers are single‐stranded oligonucleotides that achieve their remarkable binding capabilities by adopting distinctive three‐dimensional structures, including hairpins, pseudoknots, and G‐quadruplexes.^[^ ^2^, ^3^ ^]^ G‐quadruplexes, noncanonical DNA/RNA structures formed by guanine‐rich sequences, have unique biophysical properties that enhance the binding and functional versatility of aptamers, making them particularly promising for therapeutic and diagnostic applications.^[^ ^4^ ^]^ Since the advent of the SELEX (Systematic Evolution of Ligands by Exponential Enrichment) process,^[^ ^5^, ^6^ ^]^ aptamers have emerged as powerful tools for addressing unmet medical needs.

In the field of neurodegenerative disorders, aptamers offer promising avenues for tackling diseases such as Alzheimer's, Parkinson's, and Huntington's.^[^ ^7^ ^]^ By targeting pathogenic proteins, inhibiting aggregation, and modulating key pathways, they hold the potential to mitigate disease progression with precision and minimal off‐target effects. Despite their advantages, potential therapeutic oligonucleotides (TOs) face challenges such as enzymatic degradation, short circulation time, and suboptimal delivery, which can limit their clinical application.^[^ ^1^ ^]^ To overcome these limitations, innovative strategies have been developed to enhance aptamer stability and delivery.^[^ ^8^ ^]^ Indeed, incorporating protective chemical modifications can improve their resistance to degradation, while nanomaterial‐based delivery systems can transform their therapeutic potential. Nanocarriers such as halloysite nanotubes (HNTs), montmorillonite clay, and other advanced platforms offer enhanced protection, controlled release, and targeted delivery to disease sites.^[^ ^9^, ^10^ ^]^ These systems not only safeguard aptamers from enzymatic degradation but also improve their bioavailability and therapeutic efficacy.

This review examines the progress in aptamer‐based TOs, with a focus on G‐quadruplex‐forming aptamers, their application in neurodegenerative diseases, and nanomaterial‐driven delivery strategies. By exploring innovative strategies for stabilizing and delivering aptamers, we aim to highlight their potential in treating complex and otherwise intractable diseases.

## 2 Aptamers as TOs

### 2.1 Introduction to Nucleic Acid Aptamers and their Therapeutic Potential

DNA and RNA aptamers belong to the category of TOs, which, based on their different mechanisms of action in disease treatment, also include antisense oligonucleotides (ASOs), DNAzymes, immunoregulatory oligonucleotides, messenger RNA (mRNA), microRNA (miRNA), and small interfering RNA (siRNA). Aptamers are single‐stranded oligonucleotides characterized by a distinctive three‐dimensional structure that confers high affinity and specificity to their target molecules. The first aptamers were selected as far back as 1990 by three independent groups.^[^ ^5^, ^6^, ^11^ ^]^ Since then, aptamers have been extensively studied for their therapeutic and diagnostic purposes, including biosensorics applications.^[^ ^12^ ^]^ In their seminal paper, Ellington and Szostak coined the term aptamers: “We have termed these individual RNA sequences 'aptamers', from the Latin 'aptus', to fit”.^[^ ^5^ ^]^

Aptamers can bind a wide range of targets, including metal ions,^[^ ^13^ ^]^ proteins,^[^ ^14^ ^]^ viruses,^[^ ^15^ ^]^ and whole cells.^[^ ^16^ ^]^ This versatility arises from the wide variety of secondary and tertiary structures that different oligonucleotide sequences can adopt, adapting to the molecular target. Aptamers are typically identified through an iterative in vitro selection process known as SELEX,^[^ ^17^ ^]^ introduced by Tuerk and Gold.^[^ ^6^ ^]^ However, determining the three‐dimensional (3D) structure of aptamers after their identification is often a challenging task. Various complex 3D structures have been discovered, including hairpins, pseudoknots, bulges, triplexes, and G‐quadruplexes.^[^ ^18^ ^]^

Aptamer‐based therapies are being investigated for a wide range of diseases, including cancer, cardiovascular disease, autoimmune diseases, neurological, and infectious diseases.^[^ ^1^ ^]^ Aptamers often exhibit higher specificity and affinity compared to antibodies,^[^ ^19^ ^]^ and for this reason, they are often called “chemical antibodies”.^[^ ^20^ ^]^ Unlike antibodies, aptamers can be easily synthesized in large quantity at low cost, are resistant to temperature variations, and can be reversibly denatured.^[^ ^21^ ^]^ They have generally smaller size than antibody and other biomolecules utilized for therapeutic applications, resulting in rapid renal clearance and short circulating times.^[^ ^22^ ^]^ In addition, DNA‐ and RNA‐based aptamers show low immunogenicity in comparison to antibodies and other biomolecules.^[^ ^23^ ^]^

The binding affinity of aptamers to their specific target is quantified by the equilibrium dissociation constant (*K*_d_), whose value is generally nanomolar or lower. *K*_d_ can be determined using biological assays^[^ ^24^ ^]^ or directly measured by physicochemical methodologies.^[^ ^25^, ^26^ ^]^ Many different interactions occur between an aptamer and its target, particularly with proteins, such as H‐bonds, electrostatic, van der Waals, and stacking interactions. In some protein–aptamer interactions, the target protein features clefts that accommodate the aptamer and inhibit the protein activity without altering other functions.^[^ ^27^ ^]^ This ensures high specificity and minimizes off‐target effects.

As previously mentioned, despite their numerous advantages, aptamers present some significant challenges, particularly their susceptibility to degradation by nucleases and rapid renal clearance, which hinder their therapeutic effectiveness. To address these issues, various chemical modifications have been developed to enhance stability against nucleases, improve pharmacokinetics, and increase both target affinity and selectivity. In addition to chemical modifications, nanoparticles and other nonviral vectors have also been explored to enhance the stability of aptamers and efficiently deliver them to target tissues.

### 2.2 Aptamer Selection by SELEX

**Figure 1:**
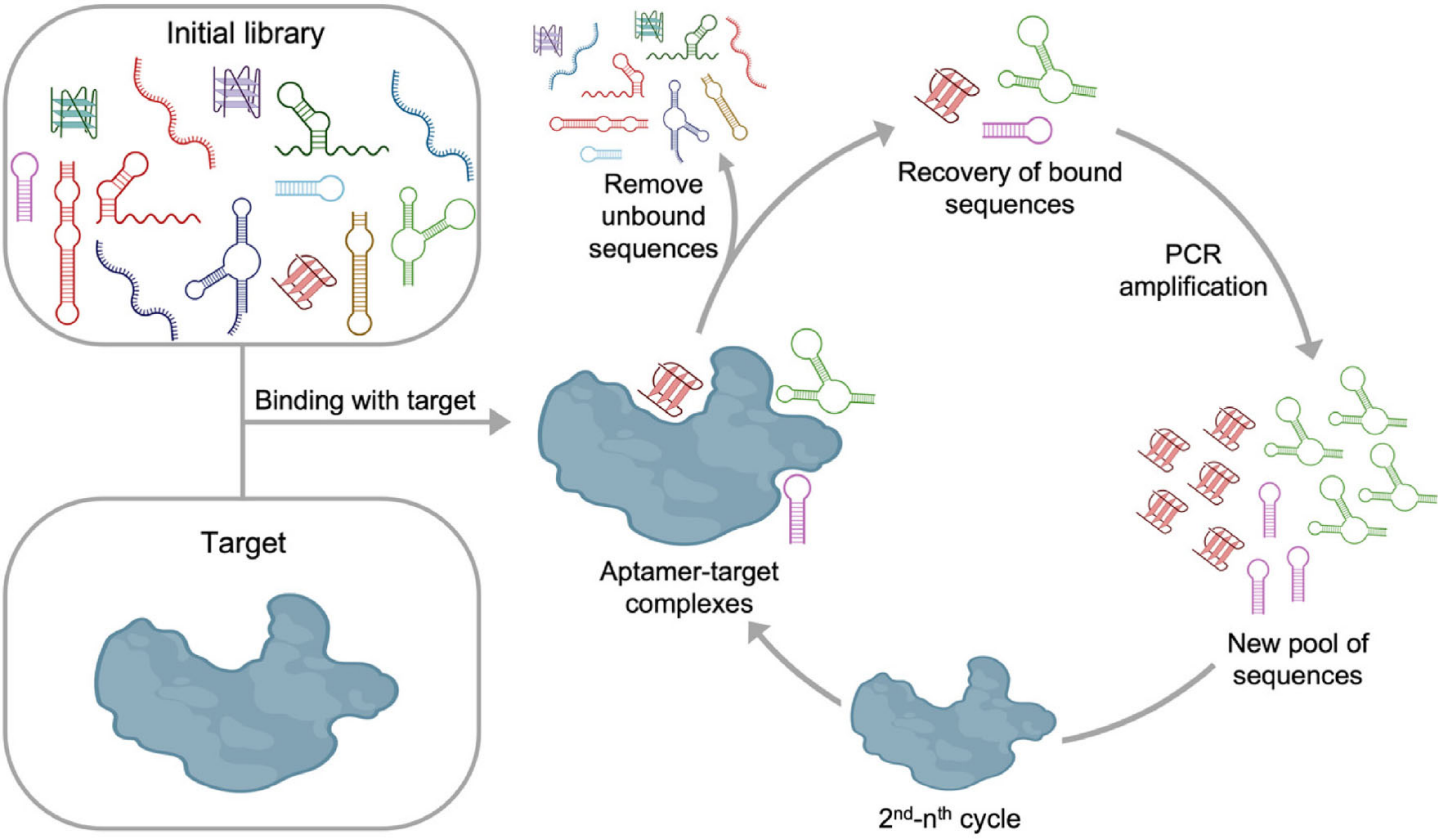
Schematic representation of the SELEX process. The initial library, containing 10^12^–10^15^ unique sequences, is incubated with the target during selection. Unbound sequences are removed, while target‐binding aptamers are recovered and amplified using PCR to generate a new pool of sequences for the next selection round. Through iterative cycles, specific aptamers are progressively enriched and finally identified by sequencing analysis. Created with BioRender.com.

As previously mentioned, aptamers are typically identified through the SELEX process.^[^ ^17^ ^]^ This process involves repeated rounds of binding, purification, and amplification to isolate high‐affinity binders for a specific target (**Figure** **1**). The initial pool consists of 10^12^–10^15^ different nucleotide sequences, each up to 100 nucleotides long and containing fixed primer regions. Upon annealing, these sequences may fold into secondary structures that may allow them to bind to targets. The SELEX process begins by incubating this diverse pool with a target of interest, which could be a protein, an organic molecule, a whole cell, or even in vivo on a live animal. To enhance specificity and minimize enrichment of off‐target binders, a negative or counter‐selection strategy is employed. During this step, the aptamer pool is first incubated with an undesired target to deplete sequences that bind to it. After counter‐selection, the “cleaned” pool, is incubated with the desired target. Unbound sequences are washed away, while bound sequences are retained, eluted, and then amplified using PCR. This amplification generates a new, enriched pool of sequences with a higher affinity for the target. In the case of RNA aptamers, a reverse transcription step is required during amplification, making the selection more tedious.^[^ ^28^, ^29^ ^]^ The cycle of binding, washing, elution, and amplification is repeated multiple times to progressively enrich the pool for high‐affinity aptamers. The resulting pool is cloned and sequenced using platforms like next‐generation sequencing. The sequences are then ranked based on their abundance in the pool. Then, top‐ranked aptamers are synthesized via solid phase chemistry and modified as needed.

Since the development of the classical SELEX method for aptamer selection, various improvements and modifications have been introduced to refine the selection process and enhance binding affinity.^[^ ^1^, ^30^, ^31^, ^32^ ^]^ Among these, magnetic‐assisted SELEX,^[^ ^33^ ^]^ cell‐SELEX,^[^ ^34^ ^]^ capillary electrophoresis SELEX,^[^ ^35^ ^]^ and automated SELEX^[^ ^36^ ^]^ are the most frequently used. In magnetic‐assisted SELEX, the target is first immobilized on the surface of magnetic beads, which are incubated with the random sequence library. Magnetic precipitation recovers bound sequences, allowing selection without sophisticated technologies and with easily tunable conditions. This method is suitable for both intracellular and extracellular proteins. However, aptamers generated via protein‐based SELEX may fail to recognize the target protein on the cell surface. Whole‐cell‐based SELEX can address this limitation, enabling the identification of aptamers that bind to the target protein in its native conformation or recognize specific glycosylation patterns, and can even facilitate internalization into target cells.^[^ ^37^, ^38^, ^39^ ^]^ To expedite high‐affinity aptamer identification, specialized partitioning technologies, such as capillary electrophoresis,^[^ ^40^ ^]^ have been successfully integrated into the classic SELEX procedure. Recent advancements have also incorporated bioinformatic analysis and high‐throughput sequencing into SELEX,^[^ ^41^ ^]^ facilitating the rapid identification of aptamers and exploring molecular evolution. Additionally, artificial intelligence (AI)‐driven machine learning (ML) for aptamer discovery offers new avenues for developing high‐quality aptamers (see Section 2.5.2).^[^ ^42^ ^]^

### 2.3 Aptamer Modifications

**Figure 2:**
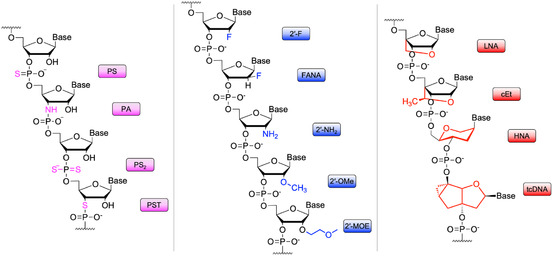
Examples of modifications used to improve the pharmacokinetic profile of aptamers by stabilizing the nucleic acids against enzymatic hydrolysis and rapid renal clearance (PS: phosphorothioate; PA: phosphoramidate; PS_2_: phosphorodithioate; PST: phosphorothiolate; 2′‐F: 2′‐fluoro; FANA: 2′‐fluoroarabino; 2′‐NH_2_: 2′‐amino; 2′‐OMe: 2′‐*O*‐methyl; 2′‐MOE: 2′‐*O*‐methoxyethyl; LNA: locked nucleic acid; cET: constrained ethyl; HNA: hexitol nucleic acid; tcDNA: tricyclo DNA).

Unmodified aptamers, particularly RNA‐based aptamers, are unstable and susceptible to degradation by nucleases in the blood, with half‐lives ranging from minutes to tens of minutes, which is inadequate for most clinical applications.^[^ ^1^, ^43^, ^44^ ^]^ To enhance nuclease resistance and binding affinity, most aptamers in clinical studies undergo chemical modifications.^[^ ^45^ ^]^ Two primary strategies are used to incorporate these modifications: in‐SELEX (or pre‐SELEX) and post‐SELEX, each offering distinct advantages and limitations.^[^ ^46^ ^]^ In‐SELEX strategy involves isolating aptamers with the desired modifications directly from a DNA or RNA library that contains compatible nucleotides. This strategy allows for modifications such as 2′‐amino, 2′‐fluoro, and 2′‐*O*‐methyl nucleotides, as well as locked nucleic acids (LNA) (**Figure** **2**). A key advantage of in‐SELEX is that it enables the selection of sequences already optimized to adopt stable conformations in the presence of modifications, reducing the risk of impairing aptamer functionality due to structural perturbations. Moreover, in‐SELEX enables the generation of highly specialized aptamers, such as SOMAmers (developed by SomaLogic), which employ modified deoxyuridine residues with hydrophobic groups at the C5 position, enhancing both stability and specificity.^[^ ^47^ ^]^

However, in‐SELEX requires polymerases capable of efficiently incorporating modified nucleotides, which limits the repertoire of possible chemical modifications and may complicate the amplification steps during selection. Additionally, mirror‐image aptamers, known as Spiegelmers, are also selected via in‐SELEX against enantiomeric targets. While they offer excellent in vivo biostability, the synthesis of the target enantiomer, required for selection, can be technically challenging and costly, limiting their practical utility.^[^ ^48^, ^49^ ^]^ In contrast, the post‐SELEX strategy involves introducing several modifications at various positions (mainly on the sugar ring or phosphate group) during the solid‐phase chemical synthesis of pre‐selected aptamers (Figure 2). This approach allows for the combination of multiple modifications to achieve optimal performance. However, introducing modifications post‐selection carries the risk of altering the aptamer's native folding or reducing its binding affinity and specificity.^[^ ^50^ ^]^ Therefore, extensive post‐modification characterization is required to verify that the modified aptamer retains its functional conformation and target recognition capability. Thus, while in‐SELEX excels in producing aptamers pre‐adapted to chemical modifications, it is limited by the chemistry tolerated during enzymatic amplification, whereas post‐SELEX provides broad modification possibilities but requires meticulous optimization to preserve biological activity.

In addition to stability issues, aptamers face challenges related to rapid clearance from the bloodstream due to their low molecular weight (5–10 kDa), which limits their therapeutic efficacy.^[^ ^51^ ^]^ To overcome renal filtration and extend circulation time, various conjugation strategies are employed, including attachment to high‐molecular‐mass polyethylene glycol (PEG), cholesterol, proteins, liposomes, and organic or inorganic nanomaterials.^[^ ^51^ ^]^ These approaches aim to increase the molecular weight of the aptamer constructs, thus preventing rapid renal clearance. Conjugation with PEG, a well‐studied hydrophilic biomaterial, improves solubility, reduces aggregation, and is widely used in FDA‐approved formulations to prolong circulation half‐life and improve in vivo bioavailability. For instance, PEGylation of the aptamer Macugen increased its half‐life to 9.3 h in plasma and to 94 h in the vitreous humor.^[^ ^52^ ^]^ Beyond extending half‐life, PEGylation also enhances the stability and reduces the immunogenicity of aptamers. In contrast, conjugation to nanomaterials can also facilitate targeted delivery and improve therapeutic efficacy.^[^ ^51^ ^]^

Multimerization is another approach that involves the incorporation of multiple aptamer units to generate multivalent molecules above the renal filtration cut‐off threshold (30–50 kDa), enhancing binding affinity, specificity, and pharmacokinetics compared to monovalent counterparts.^[^ ^51^ ^]^ For example, tetrameric aptamer conjugates have shown improved circulation retention and therapeutic potential.^[^ ^53^, ^54^ ^]^

### 2.4 Enhancing Aptamer Selection through Structured Libraries: Focus on G‐Quadruplexes

**Figure 3:**
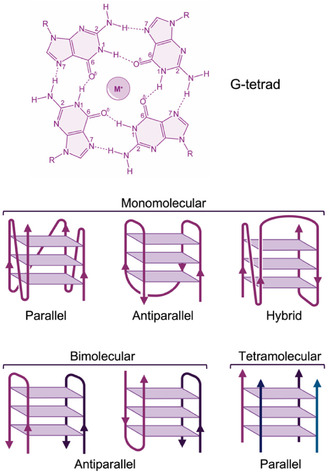
Schematic representations of (top) a G‐tetrad stabilized by a monovalent cation (M^+^) and (bottom) G‐quadruplex structures classified according to the number of strands and their relative orientation.

The selection phase is one of the most crucial steps for successful aptamer identification. A strategy to increase the chances of success involves the use of pre‐structured libraries.^[^ ^45^ ^]^ RNA aptamers inherently tend to exhibit more diverse three‐dimensional conformations and stronger intramolecular interactions, which likely enhance binding affinity and specificity compared to DNA aptamers.^[^ ^1^ ^]^ One pre‐structured library strategy involves stabilizing the aptamer structure by incorporating double‐stranded stems or junction motifs in the constant regions.^[^ ^45^ ^]^ The predictable final structure of these aptamers also simplifies the process of designing truncations.^[^ ^55^ ^]^ Another library strategy involves the use of guanine (G)‐rich sequences in the random region to increase the probability of forming G‐quadruplexes.^[^ ^56^ ^]^ These noncanonical secondary structures can generally form in both DNA and RNA strands with a consensus sequence of G_≥2_N_1–7_G_≥2_N_1–7_G_≥2_N_1–7_G_≥2_, where N represents any nucleotide.^[^ ^57^ ^]^ However, some nonconsensus sequences have also been reported to fold into stable G‐quadruplexes.^[^ ^57^ ^]^ G‐quadruplex structures are stabilized by stacks of G‐tetrads, which are square planar arrangements of four guanine bases held together by Hoogsteen hydrogen bonds (**Figure** **3**). Monovalent cations embedded in or between the G‐tetrads provide additional stability. G‐quadruplexes display substantial structural polymorphism, adopting different conformations influenced by factors such as the nucleic acid sequence, the presence of specific ions, and environmental conditions.^[^ ^58^ ^]^ These structures can differ in strand orientation (parallel, antiparallel, or hybrid), loop configuration (diagonal, lateral, or propeller), and the number of strands involved (monomolecular, bimolecular, or tetramolecular) (Figure 3). The polymorphic nature of G‐quadruplexes enables them to fulfill diverse roles in biological systems, adapting to various functional requirements and environmental conditions. This versatility makes them intriguing candidates for therapeutic and biotechnological applications, as their distinct conformations may exhibit distinct biological activities and binding properties.^[^ ^59^, ^60^, ^61^, ^62^ ^]^ G‐quadruplex structures are often present in high‐affinity aptamers.^[^ ^63^, ^64^, ^65^, ^66^, ^67^, ^68^, ^69^ ^]^ Therefore, increasing their prevalence in the initial library may improve the probability of success. Besides their unique structure, G‐quadruplexes also play important roles in crucial cellular processes, including DNA transcription, replication and repair, epigenetics, and RNA translation.^[^ ^70^, ^71^, ^72^ ^]^

### 2.5 In Silico Strategies for Aptamer Design and Optimization

#### 2.5.1 Computational Modeling of Aptamers

Computational modeling techniques, and in particular molecular dynamics (MD) simulations, are increasingly recognized as important tools in the design and characterization of therapeutic aptamers, providing detailed insights into their structural properties, conformational flexibility, and molecular interactions with biological targets. These in silico methods offer a powerful complement to experimental approaches by enabling atomic‐resolution analyses of aptamer–target interactions and supporting the rational optimization of aptamer sequences.^[^ ^73^, ^74^, ^75^, ^76^ ^]^ This is especially relevant in the field of neurodegenerative diseases, where pathogenic protein targets are typically dynamic, aggregation‐prone, and structurally complex.^[^ ^77^ ^]^

In this context, computational tools not only assist in the identification of aptamers but also aid in deciphering the mechanisms of target inhibition, facilitating the development of more effective and selective TOs.^[^ ^73^ ^]^

An example of the application of computational methodologies in aptamer research related to neurodegenerative diseases is provided by Mashima et al., who studied the antiprion RNA aptamer R12 targeting the cellular prion protein (PrP^C^) (see Section 3.1). Structural studies combined with MD simulations revealed that R12 adopts a dimeric G‐quadruplex architecture stabilized by *π*−*π* stacking of G(:A):G:G(:A):G hexad planes. Molecular modeling confirmed that this unique topology is essential for high‐affinity binding and inhibitory activity against prion conversion. The insights gained from the computational analysis of R12's G‐quadruplex structure directly guided the design of enhanced analogues such as R24 and R12‐A‐R12, which displayed improved stability and antiprion activity in prion‐infected cell models.^[^ ^78^ ^]^ The identification and characterization of such structural motifs through simulation not only elucidate aptamer function but also support the design of more robust therapeutic candidates.

Computational approaches have also contributed to aptamer studies in Parkinson's disease (PD). Rock et al. used in silico modeling to rationally redesign the α‐synuclein‐binding aptamer M5‐15, obtaining a new variant, TMG‐79, with enhanced binding affinity (see Section 3.3).^[^ ^79^ ^]^ Although full MD simulations were not performed, structural analysis suggested that the improved binding of TMG‐79 to α‐synuclein may be attributed to its increased flexibility, which facilitates more effective target recognition.

Beyond these individual case studies, computational modeling is increasingly being integrated into the aptamer discovery pipeline. Sequence‐structure prediction algorithms, molecular docking, and free energy calculations are being used to prescreen aptamer candidates and refine their interactions with protein targets. These methods are particularly powerful when studying aptamers that form G‐quadruplex structures, as their folding topology, loop orientation, and cation coordination significantly influence target binding.^[^ ^25^, ^67^ ^]^ Simulations performed under physiological ion conditions can predict the most stable conformers, which in turn correlate with functional performance.

These computational strategies also play a key role in rational sequence refinement, helping predict the effects of specific modifications on aptamer folding, stability, and interaction with targets.^[^ ^76^ ^]^ They provide mechanistic insights that guide the design of aptamers with improved biostability, target specificity, and pharmacokinetic properties. Furthermore, they reduce experimental workload by limiting the number of sequences requiring synthesis and validation, thus streamlining the optimization process.^[^ ^73^ ^]^ Advanced computational protocols, including enhanced sampling techniques, are now being applied to capture the conformational dynamics of aptamers and their targets over biologically relevant timescales.^[^ ^80^, ^81^ ^]^ These methods are particularly useful for simulating interactions with intrinsically disordered proteins, such as α‐synuclein, tau, or amyloid‐β, whose aggregation‐prone and flexible nature complicates structural characterization. In such systems, aptamers often exert their therapeutic effect by interfering with aggregation nucleation or oligomer propagation, mechanisms that can be effectively explored through long‐timescale MD simulations.

In addition to structural characterization, computational methods can assist in predicting aptamer–target binding free energies, identifying key contact residues, and simulating the effects of sequence modifications on structure and target recognition. This information enables the rational engineering of aptamers with enhanced biological function, reduced susceptibility to degradation, and minimized off‐target interactions. As therapeutic aptamers move toward clinical application, the role of computational modeling is expected to become even more central, particularly in the context of complex diseases such as neurodegenerative disorders.

#### 2.5.2 AI Approaches

AI techniques, including ML and deep learning (DL), are rapidly gaining traction as powerful complements to traditional in silico methods for aptamer design. These approaches are able to learn complex sequence–structure–function relationships from experimental datasets, enabling the prediction and generation of high‐affinity binders, significantly reducing the number of expensive laboratory rounds such as SELEX. Unlike classical computational pipelines (sequence‐structure prediction, docking, MD), AI methods can directly use sequence data to predict aptamer–target affinity, design novel sequences, and guide early‐stage selection, marking a paradigm shift in aptamer development.^[^ ^82^ ^]^

A notable example is AptaTrans, a DL pipeline that employs transformer‐based encoders to process both aptamer and protein sequences at the monomer level, integrating structural representation through pretraining.^[^ ^83^ ^]^ These encoders use self‐attention mechanisms to model long‐range dependencies and contextual features within biological sequences.^[^ ^84^ ^]^ AptaTrans predicts aptamer‐protein interaction with superior accuracy compared to conventional models and is paired with a generative algorithm (Apta‐MCTS) to propose new aptamer candidates for validation.^[^ ^85^ ^]^ By capturing contextual features from large datasets, AptaTrans anticipates optimal aptamer–target pairings, accelerating discovery and reducing false leads.

Another representative platform is AIoptamer (AI‐driven optimization of aptamers), which integrates sequence‐level DL with structure‐based modelling and MD simulations to accelerate aptamer discovery and design.^[^ ^86^ ^]^ Starting from known aptamer–target complexes, AIoptamer generates and ranks sequence variants using binding affinity predictors, followed by 3D modelling and MD‐based structural validation. This integrated AI‐driven pipeline demonstrated the ability to identify high‐affinity aptamer candidates more efficiently than traditional approaches, while also reducing reliance on extensive SELEX cycles.

Similarly, DeepAptamer integrates sequence composition and structural features to predict aptamer binding affinities and potential binding motifs.^[^ ^87^ ^]^ Trained on early SELEX round data, this hybrid model can efficiently identify high‐affinity aptamers while reducing the typical 20‐30 iterative rounds used in SELEX workflows. DeepAptamer also highlights binding motifs and key nucleotides critical for target interaction, guiding rational sequence modification.

Another ML‐based approach employs particle display to partition a library of aptamers by affinity and uses such data to train ML models to predict affinity in silico.^[^ ^42^ ^]^ The ML‐guided particle display methodology yielded significantly improved candidate yield, 11‐fold higher than random sequence perturbation, and produced novel truncated aptamers (≈30% length reduction) with higher binding affinity than the best experimental candidate.

Beyond affinity prediction, unsupervised ML method known as the Potts model has been used to diversify aptamer sequence libraries by generating novel candidates with desired secondary structures.^[^ ^88^ ^]^ Such approach expands functional chemical diversity, improving chances of identifying high‐performance aptamers. Finally, recent reviews highlight that AI‐based binding prediction models consistently outperform classical docking and screening tools, especially when large experimental datasets are available.^[^ ^89^ ^]^

AI‐driven methods now permeate multiple stages of aptamer discovery: prediction, candidate design, and structural optimization. These approaches accelerate selection, expand design possibilities, reduce experimental burdens, and enhance binding performance. As more extensive and annotated aptamer–target datasets become available, AI frameworks will likely deliver even more accurate predictions and contribute to the next generation of therapeutic and diagnostic oligonucleotides.

## 3 Aptamers in Neurodegenerative Disorders

**Figure 4:**
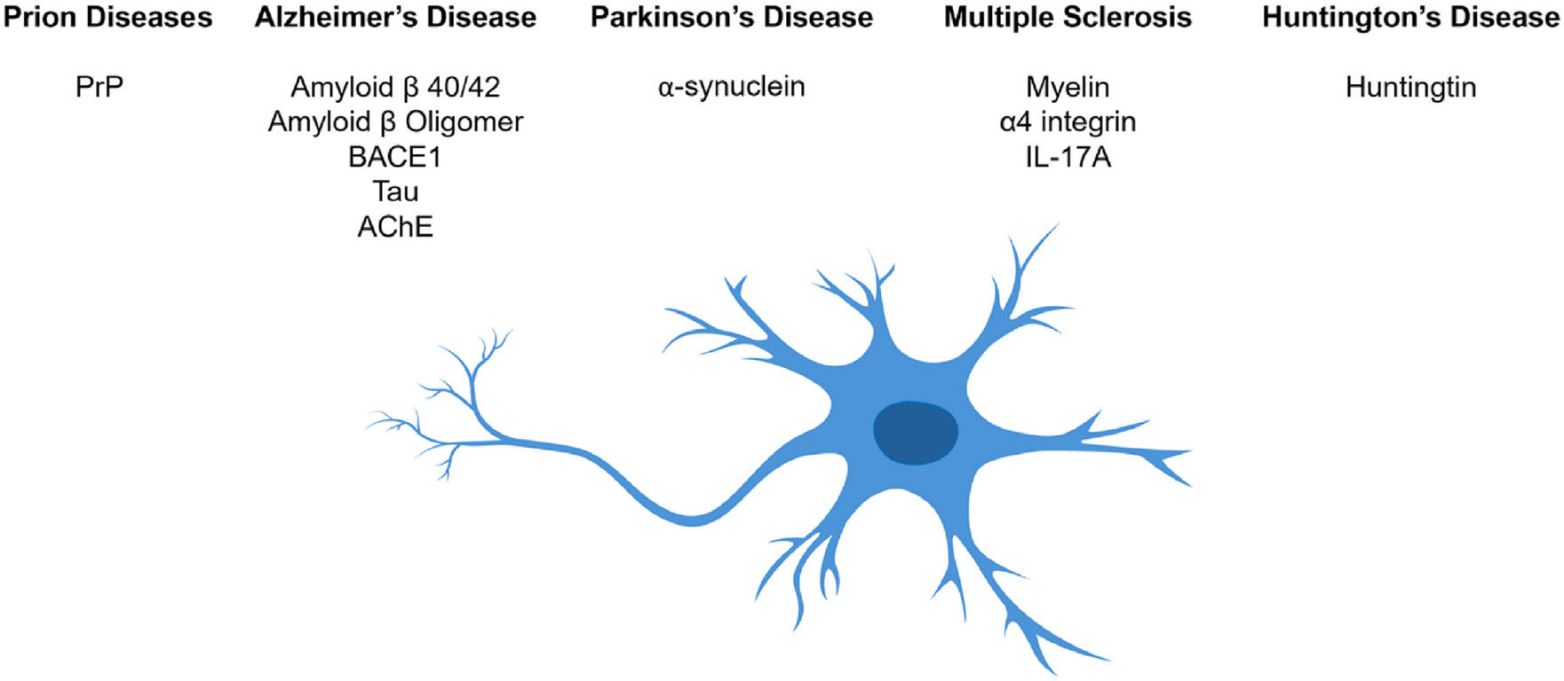
Potential aptamer targets in neurodegenerative diseases discussed in this review.

Neurodegenerative disorders are primarily characterized by the deposition of misfolded proteins, such as amyloid‐β, tau, α‐synuclein, huntingtin, and prion protein (PrP)^[^ ^90^ ^]^ (**Figure** **4**).

Therefore, preventing or reversing the deposition of these proteins could offer a promising therapeutic approach, with these abnormal proteins representing important targets for nucleic acid‐based therapeutics, such as aptamers. This section focuses on aptamers as potential therapeutic agents for neurodegenerative disorders, including prion diseases, Alzheimer's, Parkinson's, and Huntington's diseases (HDs), and multiple sclerosis (MS), where they can selectively bind pathogenic proteins, inhibit aggregation, and potentially modulate disease‐related pathways.

To contextualize the scientific relevance and evolution of this research area, a bibliometric survey of peer‐reviewed publications over the past 25 years (2000 to present) was conducted. The analysis was performed using the Scopus database, with search queries combining the term “aptamer” with each neurodegenerative disease name. The search yielded a total of 624 publications (only original research articles and reviews were considered). The annual distribution of these articles revealed a clear upward trend, with a very limited number of studies in the early 2000s (only 67 publications between 2000 and 2009), followed by a significant increase beginning around 2012 (194 publications from 2010 to 2019), and a marked acceleration in the last 5 years (363 publications from 2020 to June 2025). This trend reflects the growing scientific interest in aptamer‐based strategies for targeting disease‐related proteins and pathways in neurodegeneration.

A breakdown by disease indicates that Alzheimer's disease (AD) accounts for the largest proportion of the publications (367), followed by PD (145), prion diseases (134), MS (77), and HD (48). This distribution likely reflects the global prevalence of these disorders and the availability of well‐characterized molecular targets, such as amyloid‐β, tau, and α‐synuclein. Notably, prion diseases, although rare, have attracted significant attention in early aptamer research, especially due to the pioneering work on G‐quadruplex‐forming aptamers targeting PrP, first described in 1997.^[^ ^91^ ^]^ In contrast, aptamer applications in MS are still emerging, likely due to the complex immunopathological nature of the disease.

### 3.1 Prion Diseases

Transmissible spongiform encephalopathies (TSEs), also known as prion diseases, are a group of rare but fatal neurodegenerative disorders affecting both humans and animals.^[^ ^92^, ^93^ ^]^ These include Creutzfeldt–Jakob disease in humans, bovine spongiform encephalopathy in cattle, and scrapie in sheep.

They are characterized by the accumulation of abnormal prion protein in the brain, leading to extensive brain damage and a range of severe clinical symptoms, including rapidly progressive dementia and motor dysfunction.^[^ ^93^ ^]^

The prion protein (PrP), mainly expressed in the brain, is central to the pathogenesis of these disorders. It exists in two alternative forms: a normal, physiologically present, cellular form (PrP^C^) and a pathological conformer (PrP^Sc^). PrP^C^ is a soluble, protease‐sensitive, α‐helix‐rich isoform, while PrP^Sc^ is an insoluble, protease‐resistant, β‐sheet‐rich isoform, whose exact structure remains unknown. The pathogenesis of TSEs is linked to the conversion of PrP^C^ into PrP^Sc^. Once formed, PrP^Sc^ can propagate, recruiting more PrP^C^ to fold into new PrP^Sc^.^[^ ^94^ ^]^ This leads to an accumulation of PrP^Sc^ in tissues, causing severe cellular damage and neurodegeneration.

Therapeutic approaches for TSE are still under development. One promising strategy involves the use of aptamers that specifically bind to PrPs. These aptamers could prevent protein conversion and accumulation by stabilizing PrP^C^, inhibiting the interaction between PrP^C^ and PrP^Sc^, or blocking PrP^C^ from binding to any pathological cofactor.^[^ ^95^, ^96^ ^]^ Moreover, PrP‐specific aptamers could serve as diagnostic tools, providing a more effective way to detect and study prion diseases.^[^ ^97^ ^]^

Many studies have focused on selecting RNA and DNA molecules that can specifically bind to both PrP^C^ and its β‐rich isoform. Notably, some PrP^C^‐targeting aptamers have been shown to effectively reduce PrP^Sc^ levels in cells infected by the TSE agent, highlighting their therapeutic potential for prion diseases.^[^ ^98^, ^99^ ^]^ Noteworthy, certain aptamers with high affinity for PrP^C^ and significantly reducing PrP^Sc^ formation have been found to share common sequences and structural motifs, such as contiguous guanine repeats and G‐quadruplex structures.^[^ ^91^, ^98^, ^99^, ^100^ ^]^ Indeed, the first RNA aptamer for PrP^C^, selected by Weiss et al. using the SELEX method targeting the recombinant Syrian hamster PrP^C^, was assumed to fold into a G‐quadruplex structure, which appeared essential for protein recognition, as replacing the guanines with uridines in the aptamer sequence abolished its binding to PrP^C^.^[^ ^91^ ^]^ Notably, this aptamer specifically interacted with PrP^C^ from brain homogenates of healthy hamsters, cattle, and mice, but did not recognize PrP^Sc^ in brain homogenates from prion‐infected mice. The conservation of the specific PrP^C^–RNA interaction across different species marked a significant milestone in developing aptamer‐based tools for prion diseases.

In 2002, the same research group demonstrated for the first time the therapeutic potential of aptamers against prion diseases by selecting a 2′‐amino‐2′‐deoxypyrimidine‐modified RNA aptamer, which reduced PrP^Sc^ accumulation in prion‐infected neuroblastoma cells.^[^ ^98^ ^]^ This modification was introduced after the SELEX process to enhance RNA resistance to nucleases. Since then, numerous researchers have used the SELEX method to target either PrP^C^ or pathogenic‐related conformations.

In 2008, Murakami et al. obtained RNA aptamers against recombinant bovine PrP.^[^ ^100^ ^]^ Interestingly, all identified aptamers contained a core of four GGA tandem repeats, identified as the minimal sequence required for high‐affinity binding to the protein. This 12‐nucleotide‐long RNA molecule with the sequence r(GGAGGAGGAGGA), named R12, was recognized as one of the most effective antiprion aptamers.^[^ ^99^ ^]^ R12 folds into a peculiar G‐quadruplex structure, forming a dimeric architecture where two parallel‐stranded unimolecular G‐quadruplexes stack in a tail‐to‐tail orientation through *π*–*π* interactions of unusual G(:A):G:G(:A):G hexad planes.^[^ ^101^ ^]^ Based on the structure of R12, Mashima et al. developed two new RNA aptamers by tandemly connecting two R12 sequences: r(GGAGGAGGAGGAGGAGGAGGAGGA) (R24) and r(GGAGGAGGAGGA‐A‐GGAGGAGGAGGA) (R12‐A‐R12).^[^ ^78^ ^]^ Both aptamers adopted a unimolecular G‐quadruplex structure similar to that formed by two R12 molecules. The 50% inhibitory concentration (IC_50_) values for PrP^Sc^ formation in scrapie‐infected cell lines were ≈100 nM for R24 and 500 nM for R12‐A‐R12, representing a 100‐fold and 20‐fold reduction compared to R12, respectively. Aside from some antibodies, R24 exhibited the lowest recorded IC_50_ and the highest antiprion activity.

The DNA versions of the R12 and R24 aptamers, named D12 and D24, share several structural features with the G‐quadruplexes formed by their respective RNA counterparts.^[^ ^102^, ^103^ ^]^ Specifically, D12 forms a homodimer of intramolecular parallel‐stranded G‐quadruplexes stabilized by base‐stacking interactions between G(:A):G(:A):G(:A):G heptad planes of the two G‐quadruplexes, while D24 adopts a similar structure as a monomeric architecture. Moreover, D12 was shown to bind to PrP^C^,^[^ ^104^ ^]^ although its antiprion activity was less potent than that of R12.^[^ ^99^ ^]^

The effects of various chemical modifications aimed at enhancing the resistance of these G‐quadruplex‐forming DNA aptamers against nucleases were also investigated, since, as discussed above, improving their stability and persistence in biological fluids is crucial for their therapeutic efficacy. A small library of chemically‐modified D12 aptamers was developed, followed by physicochemical characterization and evaluation of their antiprion activity.^[^ ^26^ ^]^ These modifications included replacing the ribose 2′‐H with a methoxy group (2′‐OMe) or a fluorine atom (2′‐F) and introducing oligonucleotide end caps resulting in an inversion of polarity of the chain. Notably, all modified oligonucleotides exhibited higher activity than D12, with the 2′‐F derivatives generally outperforming the 2′‐OMe derivatives, which was consistent with their higher affinity for the protein.

### 3.2 AD

AD is the most common neurodegenerative disease, which affects 60%‐80% of people with dementia. It is characterized by a significant loss in speech expression, visuospatial processing, and executive function, along with prominent amnestic cognitive impairment.^[^ ^105^ ^]^

The pathogenesis of AD is characterized by the deposition of amyloid‐β (Aβ) plaques in the brain, caused by altered cleavage of amyloid precursor protein (APP) by γ‐ and β‐secretases (BACE1),^[^ ^106^, ^107^ ^]^ leading to the formation of insoluble Aβ fibrils. Subsequently, Aβ oligomerizes, and polymerizes into insoluble amyloid fibrils that aggregate into plaques. As a result, kinases are activated causing the hyperphosphorylation of the microtubule‐associated tau protein and its polymerization into insoluble neurofibrillary tangles. The aggregation of plaques and tangles is followed by microglia recruitment around the plaques, which increases the local inflammatory response and microglial activation, thereby increasing neurotoxicity.^[^ ^108^ ^]^

The first evidence of aptamer‐Aβ binding in Alzheimer brain tissue was given by Chakravarthy et al.^[^ ^109^ ^]^ Essentially, they developed a stable DNA aptamer with a stem‐loop structure, named RNV95, targeting low‐molecular‐weight Aβ40 oligomers.

Two years later, Murakami et al. successfully generated a series of RNA aptamers against Aβ oligomers.^[^ ^110^ ^]^ These aptamers, namely E22P‐AbD4, E22P‐AbD31 and E22P‐AbD43, showed a higher affinity for the toxic oligomeric species Aβ42 protofibrils (PFs), compared to fibrils formed by Aβ42 monomer, due to the higher affinity for the toxic dimer unit of Aβ42. Notably, E22P–AbD43 showed preferential binding to PFs, likely due to the formation of a G‐quadruplex structure, as suggested by comparing the circular dichroism (CD) absorption data of its the full‐length and random regions. Furthermore, in vitro study on SH‐SY5Y human neuroblastoma cells demonstrated the ability of E22P–AbD43 to prevent the formation of Aβ42 aggregates and their neurotoxicity.

In 2009, Laurén et al. reported that Aβ oligomers bind to prion protein (PrP^C^), which serves as a cell membrane receptor and may act as a mediator of Aβ‐oligomer‐induced synaptic dysfunction, suggesting that PrP^C^‐targeting pharmaceuticals may have therapeutic potential for AD.^[^ ^111^ ^]^ A decade later, Iida et al. presented the first example of a nucleic acid disrupting the PrP‐Aβ interaction.^[^ ^112^ ^]^ Indeed, they demonstrated that the RNA aptamer R12, by binding to PrP, can interfere with its interaction with the Aβ oligomers or monomers. Therefore, R12 may also hold therapeutic potential for AD.

In 2015, aiming of interfere with Aβ production and provide treatment for AD, Liang et al. identified the A1 DNA aptamer able to inhibit BACE1 activity, and potentially to reduce the formation of Aβ‐40 and Aβ‐42, both in vitro and in vivo.^[^ ^113^, ^114^ ^]^ Four years later, Xiang et al. developed two additional DNA aptamers against BACE1, named BI1 and BI2, that through their interactions with the β‐secretase were able to inhibit its activity, reduce Aβ levels, and restore neuronal function in cells. Furthermore, to increase their inhibitory potency, BI1 and BI2 were successfully modified by adding a cholesteryl tetraethylene glycol (TEG) moiety.^[^ ^115^ ^]^

Since dysfunctional tau protein is more strongly correlated with dementia than amyloid, targeting tau may offer greater potential for improving cognitive function in AD patients. In this regard, in 2016, Kim et al. demonstrated that human diseases caused by the oligomerization of excessive tau proteins are promising targets for inhibitory aptamers. Indeed, they identified an RNA aptamer that specifically binds to the longest isoform of human tau (tau40), inhibiting its oligomerization propensity both in vitro and in cultured cell models of tauopathy, while also potentially promoting the disassembly of accumulated tau oligomers under pathologic conditions.^[^ ^116^ ^]^

Recently, Wang et al. identified a set of aptamers targeting monomeric tau protein. Among these, BW1 showed the highest binding affinity and specificity against Tau441 and proved to inhibit oligomeric tau formation in vitro antibody assay. Afterward, to optimize the sequence without compromising affinity toward tau, the full‐length aptamer BW1 was truncated and further modified by replacing a G:T pair with an A:T pair in the stem structure to enhance stability. The resulting aptamer, BW1c, exhibited a stronger affinity for tau protein and the ability to block its oligomerization and aggregation mediated by arachidonic acid. Furthermore, BW1c showed the ability to penetrate the blood–brain barrier (BBB) and reach the neuronal cell body. Thus, this aptamer may open the door to a first‐in‐class neurotherapeutic to lessen tauopathy‐associated neurodegenerative disorders with additional development and refinement.^[^ ^117^ ^]^

The development of cognitive dysfunction in AD is closely associated with structural alterations in cholinergic synapses and reduced levels of acetylcholine (ACh). Specifically, elevated levels of acetylcholinesterase (AChE), which lead to decreased levels of ACh, are commonly observed in AD patients. Thus, AChE inhibitors (AChEIs) are widely used in AD treatment.^[^ ^118^ ^]^

In 2022, Liang et al. investigated the inhibitory effect of three DNA aptamers (Ob1, Ob2, and Ob3), previously identified by SELEX against human brain AChE,^[^ ^119^ ^]^ on the enzyme activity of mouse AChE in vitro. They also evaluated the effect of these aptamers on learning and memory abilities in a transgenic AD mouse model.^[^ ^120^ ^]^ Among the tested aptamers, Ob2 exhibited a significant inhibitory effect on both mouse and human AChE activity, and improved learning and memory dysfunction in mice, highlighting its potential as an effective AChEI for AD treatment.

### 3.3 PD

PD is the second‐most common progressive neurodegenerative disorder, affecting over 10 million people worldwide. It is clinically characterized by a broad spectrum of both motor and nonmotor symptoms, including resting tremor, bradykinesia,^[^ ^121^ ^]^ complex behavioral disorders, and cognitive dysfunction.^[^ ^122^ ^]^

**Figure 5:**
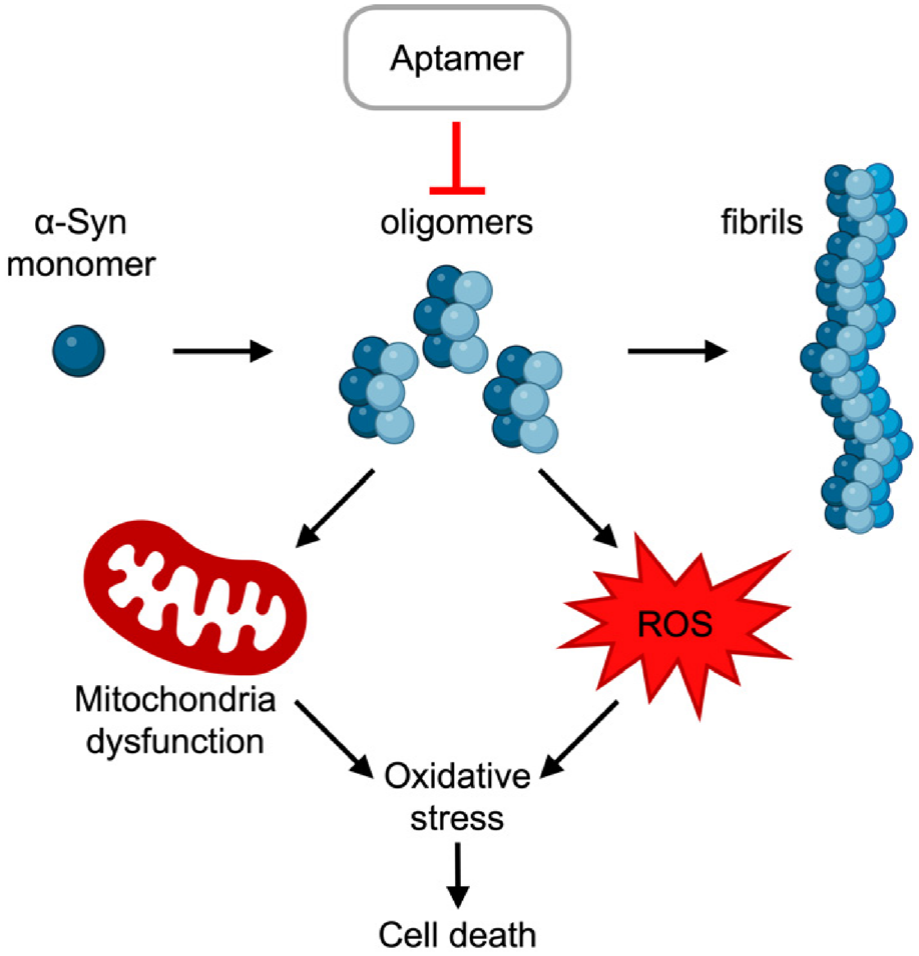
Aptamers against PD. α‐Syn oligomers disrupt the normal cellular functions, so aptamers designed to target α‐syn oligomers could serve as valuable tools for diagnosing PD or preventing its progression.

The pathogenesis of PD is typified by the degeneration of dopaminergic neurons in the substantia nigra pars compacta, along with the formation and deposition of Lewy bodies (LBs) in neurons.^[^ ^123^ ^]^ The main component of LBs is the presynaptic protein α‐synuclein (α‐syn) which is misfolded and aggregated.^[^ ^124^ ^]^ The α‐syn protein can assume different conformational states depending on conditions and cofactors, these include the helical membrane‐bound form, a partially‐folded state that is a key intermediate in aggregation and fibrillation, various oligomeric species, and fibrillar and amorphous aggregates.^[^ ^125^ ^]^ Most research outcomes support the hypothesis that α‐syn oligomers, rather than fibrils, are the primary neurotoxic form, responsible of the impairment of the degradation systems, the damage of mitochondria, the generation of oxidative stress and toxicity, culminating in neuronal death (**Figure** **5**).^[^ ^126^ ^]^ Notably, the aggregation of α‐syn is known to be accelerated by its C‐terminal truncations. However, recent structure‐activity analyses, bioinformatic studies, and NMR investigations have also suggested an important role for the N‐terminus in modulating the α‐syn aggregation.^[^ ^127^, ^128^ ^]^ To counteract these pathological mechanisms, several aptamers have been developed to reduce α‐syn aggregation in PD pathology.

The first group of aptamers targeting α‐syn was developed by Ikebukuro et al.^[^ ^129^ ^]^ Within this group, the aptamer M5‐15 demonstrated the highest affinity for α‐syn. M5‐15 recognized the amino acid sequence of α‐syn, being able to bind to both α‐syn monomers and oligomers, with a higher apparent affinity for the oligomers. In 2021, Rock et al. refined the M5‐15 structure by using an in silico approach, generating a new aptamer, named TMG‐79, which exhibited enhanced affinity for α‐syn compared to M5‐15, attributed to a greater potential flexibility.^[^ ^79^ ^]^ In 2012, Ikebukuro et al. developed eight 24‐nucleotide‐long aptamers (T‐SO517, T‐SO606, T‐SO554, T‐SO530, T‐SO552, T‐SO504, T‐SO508, and T‐SO602) that appeared to be “conformation‐specific”, binding specifically to α‐syn oligomers rather than monomers or fibrils.^[^ ^130^ ^]^ Although these aptamers did not share common sequences, their high guanine content allowed them to form G‐quadruplex structures, which may contribute to their affinity for α‐syn. Interestingly, these aptamers recognized the β‐sheet structure and could bind not only to α‐syn oligomers but also to amyloid‐β oligomers, which are associated with the development of AD. In follow‐up studies, the same group reported that stabilizing folded G‐quadruplex structures in DNA aptamers enhanced their binding properties.^[^ ^131^ ^]^ G‐quadruplex stabilizers, such as TMPyP4 and L1H1‐7OTD, improved the binding of T‐SO530 to α‐syn oligomers, suggesting that such stabilizers might be promising modulators for applications of aptamers in neurodegenerative diseases.

In 2018, Zheng et al. developed two more DNA aptamers targeting α‐syn. These 58‐nucleotide‐long DNA aptamers, F5R1 and F5R2, showed high affinity and specificity for binding to the N‐ and C‐termini of monomeric α‐syn.^[^ ^132^ ^]^ F5R1 and F5R2 aptamers inhibited the α‐syn aggregation in vitro and in primary neurons, promoting its clearance and rescuing the mitochondrial dysfunction and cellular defects caused by α‐syn overexpression. To improve intracellular delivery, the aptamers were modified by attaching a secondary amphipathic peptide, called CADY, which increased their cell membrane permeability. To enhance their therapeutic efficacy, F5R1 and F5R were encapsulated into the rabies viral glycoprotein (RVG)‐exosome and delivered into neurons, significantly reducing α‐syn aggregate levels.^[^ ^133^ ^]^

In 2022, Hmila et al. identified a group of aptamers specifically targeting the fibrillar forms of C‐terminally truncated α‐syn, and with no cross‐reactivity toward the generic β‐sheet structure found in the amyloid fibrils. The CD spectra of two aptamers, Apt11 and Apt15, agreed with a B‐DNA structure. Notably, Apt11 showed clear structural changes upon binding to fibrils of truncated α‐syn forms. Conversely, no conformational change was detected for Apt15. Moreover, Apt11 was shown to reduce the insoluble phosphorylated form of α‐syn at Ser‐129 in the cell model and to inhibit α‐syn aggregation.^[^ ^134^ ^]^

Although the precise pathogenesis of PD remains unclear, increasing evidence suggests a link between PD and oxidative stress. Numerous studies indicate that oxidative stress resulting from an excessive accumulation of reactive oxygen species plays a significant role in the neurodegeneration of dopaminergic neurons.^[^ ^135^, ^136^, ^137^ ^]^ While ascorbic acid is known to protect cells from damage caused by free radicals, it is easily oxidized and rapidly loses its antioxidant activity. To overcome this obstacle, Song et al. developed a single‐stranded DNA aptamer, NXP031, which was able to bind to ascorbic acid, reducing its oxidation and increasing its efficacy.^[^ ^138^ ^]^ In vivo experiments showed that NXP031 treatment significantly improved motor deficit and protected the nigrostriatal dopaminergic system from oxidative stress by increasing plasma ascorbic acid levels. Furthermore, in subsequent studies, the same research group demonstrated the neuroprotective effects of NXP031 by preventing dopaminergic neuronal loss in the substantia nigra and inhibiting the propagation of α‐syn pathology into the other brain regions through the reduction of oxidative stress.^[^ ^139^ ^]^

### 3.4 MS

MS is a chronic autoimmune‐mediated illness of the central nervous system (CNS), commonly resulting in progressive impairment to varied degrees. MS is one of the most common causes of neurological disability in young adults.^[^ ^140^ ^]^ The exact etiology of MS remains unclear. This disease is characterized by neurodegeneration, demyelination, oligodendrocyte loss, and axonal damage.^[^ ^141^ ^]^ The neurodegenerative process in MS can progress through both innate and adaptive immune responses, involving the activation of specific toll‐like receptors and increasing cytokine production.^[^ ^142^ ^]^ The most prominent manifestation of MS is the formation of demyelinated plaques, resulting from a combination of factors, the most important of which are activated autoreactive CD4^+^ T cells, pro‐inflammatory cytokines, and autoantibodies.^[^ ^143^ ^]^ Immunosuppressive therapies, often involving the use of monoclonal antibodies, are the most frequently used approach to block disease progression. Although generally effective, these treatments have several limitations, such as immunogenicity, sensitivity to temperature and pH, and high production costs, which can hinder their use and availability to patients.^[^ ^144^ ^]^ Consequently, many alternative therapeutic approaches are being explored, with aptamers emerging as a promising new therapeutic tools.

In 2012, Nastasijevic et al. reported the application of SELEX using a crude murine myelin suspension as a selection target to yield a myelin‐binding DNA aptamer, and identified a 40‐nucleotide‐long aptamer (LJM‐3064).^[^ ^145^ ^]^ Interestingly, multivalent streptavidin conjugates of biotinylated LJM‐3064 have been shown to rapidly distribute to the CNS and other tissues in mice, promoting remyelination of CNS lesions after intraperitoneal injection.^[^ ^145^, ^146^ ^]^ In 2013, Smestad and Maher demonstrated that LJM‐3064 folds into a G‐quadruplex motif and, under ionic conditions mimicking blood plasma, adopts a parallel G‐quadruplex conformation, likely representing its physiologically active form.^[^ ^147^ ^]^ Based on this, in 2019, Wilbanks et al. applied a rational approach to truncate LJM‐3064 to a 20‐nucleotide minimal myelin‐binding sequence (LJM‐5708).^[^ ^148^ ^]^ Myelin‐binding assay, CD spectroscopy, and gel electrophoresis experiments confirmed that LJM‐5708 retains the parallel G‐quadruplex structure of its parent aptamer, which is crucial for binding, while exhibiting significantly enhanced myelin‐binding affinity. As the DNA aptamer formulation for in vivo remyelination therapy required conjugation to streptavidin, they also verified that LJM‐5708 maintained its myelin‐binding properties in streptavidin conjugates.^[^ ^148^ ^]^ Consequently, the DNA aptamer LJM‐5708 emerged as a promising candidate for additional preclinical studies, including pharmacokinetic evaluation and in vivo remyelination experiments.

Another promising target for the development of drugs against MS is the α4 integrin. Studies have shown that antagonistic drugs of some α4 integrins can inhibit the leukocyte recruitment in MS.^[^ ^149^ ^]^ Based on the importance of α4 integrin in MS pathogenesis, a α4 integrin‐blocking antibody, natalizumab, was approved for the treatment of MS patients in 2006.^[^ ^150^ ^]^ In 2019, Kouhpayeh et al. conducted a study to discover aptamers capable of binding α4 integrin as potential alternatives to natalizumab,^[^ ^151^ ^]^ and selected a series of α4 integrin aptamer candidates that were categorized into three main clusters based on common structural motifs. Among them, the most stable aptamer, C‐12, showed a favorable dissociation constant (*K*_d_), demonstrating high affinity for α4 integrin. Future research should aim to demonstrate the efficacy of these aptamers in reducing plaque formation.

Additionally, another therapeutic strategy for the treatment of MS consists in the development of drugs able to avoid the abnormal expression of interleukin 17A (IL‐17A), a cytokine involved in this type of disorders.^[^ ^152^ ^]^ In 2022, Shobeiri et al. introduced two DNA aptamers, M2 and M7, which exhibited strong affinity for IL‐17A, with a *K*_d_ comparable to that of secukinumab, an anti‐IL‐17A monoclonal antibody. Interestingly, these two aptamers showed a synergistic effect in blocking IL‐17A as the action of the two aptamers together was more effective than that of the two aptamers alone. Finally, they selected a new small DNA aptamer with a good inhibitory effect on IL‐17A (85% blocking efficacy). Therefore, these aptamers may represent a promising therapeutic alternative to suppress and block the inflammatory cytokine IL‐17A.^[^ ^153^ ^]^

### 3.5 HD

HD is a rare, autosomal dominant inherited neurodegenerative disorder characterized by motor, cognitive, and psychiatric symptoms.^[^ ^154^ ^]^ It is caused by an abnormal expansion of a polynucleotide repeat (typically CAG) in the first exon of the gene that encodes a protein called huntingtin (HTT).^[^ ^155^ ^]^ This abnormal repetition results in the production of a mutant, misfolded HTT protein (mHTT) featured by a long polyglutamine (polyQ) tract. This polyQ extension promotes protein aggregation, especially in the caudate nucleus and putamen of basal ganglia, causing cortico‐striatal dysfunction and degeneration.^[^ ^156^ ^]^ Despite considerable advances in HD research, unfortunately, no definitive treatment is available for this disabling disease. Differences between normal and mutant huntingtin could be effectively exploited to selectively target and modulate the mutant protein, with oligonucleotide‐based aptamers providing a promising approach to slow down the disease progression.^[^ ^157^ ^]^

Over the past decade, some aptamers capable of selectively recognizing mHTT and therefore capable of inhibiting its aggregation have been studied. In 2015, Chaudhary et al. reported RNA aptamers capable of inhibiting mHTT aggregation in vitro and in a yeast model of HD by specifically binding the N‐terminal fragment of mHTT containing expanded polyQ tracts (harboring 51 or 103 glutamine residues). These aptamers were successful in reducing intracellular oxidative stress while also reducing membrane permeabilization. Additionally, they prevented the sequestration of GAPDH, a vital cellular enzyme.^[^ ^156^ ^]^ In 2018, Patel et al. also demonstrated that the same RNA aptamers were able to reduce the high calcium level resulting from the aggregation of N‐terminal mHTT with expanded glutamine repeat. These results highlighted the critical role of mitochondrial damage in the development of HD and the potential advantages of aptamers in reducing this key characteristic of the disease.^[^ ^158^ ^]^ Recently, Jane and Roy studied the effect of one of these aptamers on mHTT aggregation in both neuronal and non‐neuronal cells, advancing the understanding of its therapeutic potential.^[^ ^159^ ^]^

In 2018, through a SELEX procedure, Shin et al. identified four aptamers (MS1, MS2, MS3, MS4) capable of preferentially binding mHTT with an expanded 78‐residue polyQ tract (Q78‐HTT).^[^ ^160^ ^]^ Interestingly, these aptamers contained a high percentage of guanine nucleotides in their sequences, and secondary structure analysis using thioflavin T (ThT) fluorescence assay indicated their ability to form G‐quadruplex structures. This set of DNA aptamers forming intramolecular G‐quadruplexes, therefore, revealed a structural motif that enables preferential binding to Q78‐HTT, effectively distinguishing the elongated polyglutamine tract of mHTT from the normal HTT variant, which has a polyglutamine segment within the regular range (23 glutamine residues). Among the four investigated oligonucleotides, MS3 proved to be the strongest binder for mHTT. Based on this finding, in 2022, Riccardi et al. investigated the physicochemical properties and the biological activity of the MS3 aptamer both in vitro and in vivo.^[^ ^161^ ^]^ Results of UV, CD, and differential scanning calorimetry studies confirmed that this aptamer adopts a very stable parallel G‐quadruplex structure with strong resistance to nuclease digestion under pseudo‐physiological conditions. Moreover, MS3 exhibited rapid internalization in neuronal cells in a dose‐dependent manner. Finally, its efficacy in vivo was also demonstrated using a *Drosophila melanogaster* model expressing mHTT, where MS3 was shown to ameliorate neuronal dysfunctions associated with HD.^[^ ^161^ ^]^ With the aim of obtaining the minimal effective oligonucleotide sequence, Riccardi et al. also designed two truncated derivatives of MS3 (MS3‐17 and MS3‐33) and assessed their physicochemical and biological behavior.^[^ ^162^ ^]^ Biophysical experiments have demonstrated similar and, in some cases, improved stability and binding properties for the truncated aptamers compared to the parent molecule. Furthermore, biological assays indicated that MS3 analogs are rapidly internalized by neuronal cells, without signs of cytotoxicity. Finally, the results of in vivo tests on *Drosophila melanogaster* model demonstrated that the shorter aptamers have a better bioactivity than MS3. Overall, these studies demonstrate that the use of aptamers has high potential in anti‐HD therapeutic approaches based on specific targeting of mHTT.

### 3.6 Diagnostic Applications of Aptamers in Neurodegenerative Diseases

Beyond their role as therapeutic agents, aptamers have been extensively investigated as powerful diagnostic tools due to their high affinity and specificity for molecular targets. Their ability to selectively recognize disease‐associated biomarkers with high precision has enabled the development of highly sensitive and specific diagnostic strategies across diverse biomedical fields, including infectious diseases,^[^ ^163^ ^]^ cancer,^[^ ^164^ ^]^ cardiovascular disorders,^[^ ^165^ ^]^ metabolic syndromes,^[^ ^166^ ^]^ and neurological diseases.^[^ ^167^ ^]^ The remarkable versatility of aptamers in binding a wide range of targets, coupled with their high chemical stability, minimal immunogenicity, and ease of chemical modification, makes them ideal candidates for next‐generation diagnostic systems.^[^ ^168^ ^]^ In this context, aptamers are increasingly employed as molecular recognition elements in biosensors, so‐called aptasensors, often replacing traditional antibodies due to their superior reproducibility, adaptability, and small size, which allows for higher surface density and reduced sample volume requirements. Aptasensors couple specific target recognition with quantifiable outputs, typically optical, electrochemical, or colorimetric, enabling accurate and real‐time detection of a wide range of analytes.

Several aptamers have been developed for the ultrasensitive detection of biomarkers associated with neurodegenerative diseases, providing promising tools for early diagnosis and effective disease monitoring. A key milestone in this field was reported in 2004, when 2′‐fluoropyrimidine‐modified RNA aptamers were isolated for their ability to selectively bind β‐sheet‐rich, pathogenic forms of PrP implicated in prion diseases. In subsequent studies, the minimal binding regions of selected aptamers were identified, and their secondary structures were characterized using computational modeling and solution‐based probing. Strategic biotinylation of one of the truncated aptamers enabled its application in detecting abnormal PrP conformers in vitro.^[^ ^169^ ^]^

In 2008, a fluorescently labeled aptamer (F29‐mer) was successfully integrated into an affinity probe capillary electrophoresis platform for the sensitive and quantitative detection of thrombin, a protein involved in various pathological conditions, including AD.^[^ ^170^ ^]^ This approach demonstrated robust analytical performance in both buffer and 5% v/v human serum, highlighting its applicability in complex biological matrices. To detect tau protein, an established biomarker of AD and other tauopathies, a single‐stranded DNA aptamer with a nanomolar *K*_d_ was selected using kinetic capillary electrophoresis. The aptamer showed isoform‐selective binding to tau‐381 and tau‐410, two major variants of the protein, and was successfully employed in a sandwich‐type assay, achieving high analytical performance even in complex biological samples such as human plasma.^[^ ^171^, ^172^ ^]^ Following biotinylation, it was integrated into biosensor platforms capable of detecting tau at low picomolar concentrations. This biosensor demonstrated high selectivity, reproducibility, and stability even in complex biological matrices such as fetal bovine serum, suggesting their strong potential for clinical applications in early‐stage diagnostics.^[^ ^173^, ^174^ ^]^

Aptamers have also emerged as promising tools for molecular imaging. Their small size, rapid tissue diffusion, and ease of chemical labeling allow for selective target visualization with minimal background interference. In AD research, for instance, a multifluorescein‐labeled RNA aptamer (β55) was developed to target amyloid‐β plaques. This aptamer demonstrated target binding in ex vivo human brain tissue and in vivo in a transgenic mouse model, supporting the feasibility of aptamer‐based neuroimaging strategies.^[^ ^175^ ^]^

Efforts to detect α‐syn oligomers, pathological markers of PD, led to the development of the aptamer T‐SO517, capable of recognizing oligomeric forms of the protein during in vitro fibrillation (see Section 3.3). This sequence was incorporated into various sensing platforms, including gold nanoparticle‐based colorimetric assays, surface plasmon resonance, and electrochemical impedance spectroscopy, demonstrating its ability to detect α‐syn oligomers with high sensitivity and cost‐efficiency, thereby offering a promising strategy for PD diagnostics.^[^ ^176^ ^]^ Additionally, dopamine detection, relevant in PD diagnostics, has also been explored using aptamer‐based platforms. Fluorescent aptasensors have been developed by labeling ribonucleopeptide aptamers with fluorophores, enabling in vitro ratiometric detection of dopamine.^[^ ^177^, ^178^ ^]^ In 2012, a label‐free electrochemical aptasensor have been developed for dopamine detection in human serum, showing high sensitivity and specificity.^[^ ^179^ ^]^ Despite its advantages, electrochemical dopamine analysis is often hindered by interference from endogenous compounds. To address this, an RNA aptamer‐based biosensor was subsequently optimized to selectively recognize dopamine in the presence of other neurotransmitters.^[^ ^180^ ^]^ Further technological advancements led to the development of a nanoelectronics aptamer‐based biosensor using an array of silicon nanowire field‐effect transistors, capable of detecting extremely low dopamine concentrations with high accuracy.

A further advancement in aptamer‐based diagnostics for neurodegenerative diseases was reported in 2024, with the development of an electrochemical aptasensor for the quantification of myelin basic protein in cerebrospinal fluid.^[^ ^181^ ^]^ This sensor employs screen‐printed carbon electrodes modified with graphene oxide and functionalized with the LJM‐5708 aptamer (see Section 3.4), enabling label‐free, selective detection of myelin basic protein in complex biological samples. The platform exhibited high specificity, offering a cost‐effective and simplified alternative to conventional antibody‐based methods.

## 4 Clay Nanoparticles as Carriers for TOs

Nowadays, TOs are among the most promising drug candidates for targeted therapy due to their high specificity and ability to modulate cellular pathways that are difficult to target with traditional drugs.^[^ ^182^ ^]^ However, efficiently delivering TOs to targets remains a major challenge. Nucleic acid drugs face significant barriers to reach their pharmacological targets, including cell membranes and the BBB, which represents a major obstacle to the delivery of therapeutic aptamers to the brain. Given the physicochemical properties of unmodified aptamers, such as their negative charge, lipophobic nature, and relatively large molecular weight, most aptamers cannot effectively cross the BBB.^[^ ^183^ ^]^ Moreover, as previously mentioned, they are highly vulnerable to degradation by nucleases.

To address these limitations, the development of carriers that encapsulate, protect, and deliver TOs may be critical to their successful applications. In this context, ASOs have been able to enter clinical trials for HD treatment thanks to advances in delivery methods and improvements in CNS targeting.^[^ ^184^ ^]^ Future developments aim to enhance TO delivery to target cells, including the use of lipid‐ or polymer‐based nanocarriers. In this scenario, multimeric antibody‐oligonucleotide conjugates have been developed to target multiple receptors for enhanced specificity while simultaneously delivering different ASOs.^[^ ^185^ ^]^ Results indicate that this platform holds potential for delivering various TOs to the same cell, enabling targeted therapy.

In recent decades, significant progress has been made in developing nanoparticle‐based delivery systems for nucleic acid drugs. Nanoparticles and viruses have emerged as effective carriers, safeguarding nucleic acids from degradation while enhancing their stability, transfection efficiency, and safety. These vectors must be safe, efficient, specific, biocompatible, and easily produced to ensure the successful delivery of nucleic acid drugs to target cells. Commonly used vectors include viral vectors (such as adenovirus and retrovirus),^[^ ^186^, ^187^, ^188^ ^]^ as well as organic and inorganic carriers (such as metal‐organic frameworks, carbon nanotubes, and mesoporous silicon).^[^ ^189^, ^190^, ^191^ ^]^ Compared to viral vectors, nonviral vectors offer several advantages: they are cheaper, easier to manufacture and storage in large quantities, safer, more stable, and suitable for repeated administration.^[^ ^192^ ^]^ Nanoparticles, ranging in size from 10 to 1000 nm, are particularly favored in nonviral vector delivery studies due to their enhanced permeability and retention.^[^ ^193^ ^]^ Additionally, they exhibit longer blood circulation times, a higher surface area‐to‐volume ratio, and adjustable surface charges, making them a promising option. Examples of organic materials used as vectors include also liposomes,^[^ ^194^, ^195^ ^]^ as well as chitosan and its derivatives.^[^ ^196^, ^197^ ^]^ In this regard, innovative nanocarriers are being designed for targeted brain delivery, leveraging alternative administration routes to improve brain delivery and bypass the BBB. A multifunctional nanocarrier has been designed for AD treatment using intranasal administration.^[^ ^198^ ^]^ This approach enabled efficient drug delivery directly to the brain via the nose‐to‐brain pathway. The nanocarrier was developed to reduce Aβ deposition and stimulate autophagy and it consisted of dendrigraft poly‐_L_‐lysine (DGL) nanoparticles modified with KLVFF peptide and *Aleuria aurantia* lectin, co‐loaded with siRNA and rapamycin, providing a potential intranasal path for combination therapy of AD.

Functionalized multiwalled carbon nanotubes (MWNTs) demonstrated an intrinsic ability to cross the BBB in vitro and in vivo acting as a brain delivery nanocarrier with a brain‐targeting ligand Angiopep‐2 (ANG) onto its surface. Chemically‐functionalized MWNTs showed improved uptake in glioma brain compared to the nontargeted conjugate, supporting the suitability of ANG‐conjugated MWNTs for effective drug delivery to brain tumors.^[^ ^199^ ^]^ In contrast, commonly used inorganic materials for vectors include gold nanoparticles, silica nanoparticles, and nanoclay, which are among the most widely utilized and representative options. Layered double hydroxides (LDHs) clay have been widely recognized as effective carriers for transporting biomolecules such as siRNA.^[^ ^200^ ^]^ LDH nanoparticles can be synthesized using a co‐precipitation‐hydrothermal method and they acquire a positive surface charge by substituting a divalent cation with a trivalent cation in their lattice, allowing them to bind anionic nucleic acids. Their biocompatibility and low toxicity make them a promising carrier, while their ability to protect against environmental degradation extends TOs persistence. Additionally, they exhibit good colloidal behavior for stable dispersions and enhance the stability and release of TOs through interactions with ions in the environment.^[^ ^201^ ^]^

**Figure 6:**
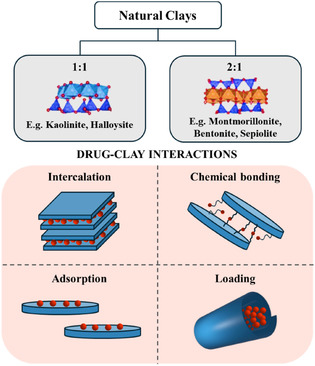
Schematic illustration of natural clay classifications and drug–clay interactions.

Here, we focus on clay nanoparticles as versatile carriers for delivering aptamers and, more generally, TOs. They have emerged due to their unique properties and large surface area, which makes them suitable for various applications. Indeed, clay nanoparticles are employed in various fields such as environmental science,^[^ ^202^, ^203^ ^]^ catalysis,^[^ ^204^, ^205^ ^]^ biomedical applications, and cultural heritage.^[^ ^206^ ^]^ As inorganic materials, clays are distinguished by interesting morphology and chemical characteristics. Clay minerals can be classified into two major categories: 1:1 and 2:1 phyllosilicates. The 1:1 phyllosilicates, such as kaolinite and HNTs, consist of one tetrahedral sheet and one octahedral sheet per clay layer (T‐O type). In contrast, 2:1 clay minerals have a structure in which each layer contains one octahedral sheet sandwiched between two tetrahedral sheets 1 (T‐O‐T type). Examples of 2:1 clay minerals include montmorillonite (MMT), laponite, sepiolite, and illite. Beyond their traditional roles as pharmaceutical excipients, clays are exploited in a wide range of pharmaceutical functions across solid (e.g., diluents, disintegrants, binders), liquid (e.g., emulsifiers, suspending agents, isotonic agents), and semisolid dosage forms (e.g., thixotropic and intumescent agents, dyes, flavoring agents, alkalizing agents), and are also increasingly applied for therapeutic purposes. The interactions between drugs and clay minerals are influenced by the physicochemical properties and functional groups of the biomolecules, as well as the reactive sites on phyllosilicate structures.^[^ ^207^ ^]^ Based on these interactions, drug‐clay hybrids can form through various mechanisms: the drug may be adsorbed onto the clay surface, be covalently bound, be intercalated between clay layers, or become entrapped within the clay's three‐dimensional network (**Figure** **6**).

One of the most attractive nanoclays is halloysite. The most appealing feature of HNT is its inner lumen, with a diameter suited to encapsulate chemical agents such as drugs, DNA/RNA, proteins, nanodots, and other active compounds.^[^ ^208^ ^]^ In this regard, the hollow lumen of halloysite acts as a miniature reservoir, supporting processes that benefit from sustained molecular release. Typically, the increase in negative surface charge within the complex further confirms the presence of a negatively charged drug inside the positively charged lumen of the nanotube, making this phenomenon easily detectable.

Recently, nano‐sized clay minerals have gained significant attention as potential carriers for nucleic acid‐based therapeutics.^[^ ^209^ ^]^ By binding and adsorbing TOs onto clay minerals, these carriers create structurally stable delivery systems that can withstand the stomach's highly acidic environment and protect TOs from enzymatic degradation before reaching target areas. Additionally, they enable the controlled release of high concentrations of TOs directly at the desired sites, significantly enhancing their therapeutic effectiveness.

Among natural clays, montmorillonite is particularly preferred for its capacity to intercalate drugs within its interlamellar spaces, a process primarily driven by ion exchange that supports a controlled release of the drug from the hybrid complex.^[^ ^210^ ^]^ The abundance of montmorillonite on early Earth attracted research into its role in prebiotic reactions, with oligonucleotides adsorption onto MMT considered significant for understanding the origins of life.

Although several models for DNA adsorption on MMT have been proposed, a general agreement on the exact mechanism has not yet been reached. Nevertheless, Wang et al. demonstrated that polyvalent metal ions facilitate oligonucleotides adsorption through a cation‐bridge model.^[^ ^211^ ^]^ In addition to electrostatic forces and hydrogen bonding, ligand exchange and cation bridging play key roles in oligonucleotides adsorption by clay minerals.^[^ ^212^ ^]^ In some cases, molecules adsorb in interlayer channels, forming cation bridges between the negatively charged silica layer on the clay surface and the phosphate groups, with exchangeable cations as intermediaries.^[^ ^213^ ^]^ However, oligonucleotides primarily adsorb on the edges or planar surfaces rather than intercalating deeply into clay layers, relying on ligand exchange, electrostatic forces, and hydrogen bonding.^[^ ^214^ ^]^ Large biomolecules generally adsorb onto planar surfaces and broken edges of clay minerals. Here, Al^3+^ coordinated with water creates acidic binding sites, promoting the binding of negatively charged substances. Both montmorillonite and kaolinite have amphoteric hydroxyl groups on their broken surfaces, enabling hydrogen bonding with polar DNA molecules, particularly on edges and corners.^[^ ^215^ ^]^ In the following sections, we focus on the systems that include both intercalation and modification of nanoclays for TO delivery.

### 4.1 Intercalated Nanoclays for TOs Delivery

Kaolinite has several pharmaceutical applications, serving both as an active ingredient and an excipient. It is also used as a delivery system for anticancer drugs, such as doxorubicin (DOX).^[^ ^216^ ^]^ Zhang et al. developed a bifunctional kaolinite‐based system to enhance the efficacy of DOX administration while simultaneously reducing its toxicity in treating thyroid cancer.^[^ ^217^ ^]^ In this system, the basal interlayer spacing of kaolinite was expanded by intercalating methoxy groups, thereby increasing its drug‐carrying capacity and enabling for controlled drug release. The structure and properties of silicate clays are crucial for achieving efficient nucleic acid loading capacity, directly affecting the extent of their adsorption. For instance, Cai et al. compared the nucleic acid binding capabilities of montmorillonite and demonstrated that montmorillonite has a stronger binding affinity.^[^ ^218^ ^]^ Additionally, the structure and molecular weight of the nucleic acids themselves significantly affect binding capacity; lower molecular weight nucleic acids generally enhance the adsorption capacity of silicate clays such as montmorillonite and kaolinite.

Comparative studies on the abilities of both kaolinite and halloysite (HNT) to bind large and small nucleic acids have highlighted key differences and similarities in their binding behaviors.^[^ ^219^ ^]^ Notably, vacuum treatment has been shown to enhance the binding of small nucleic acids to halloysite, an effect further amplified by the addition of divalent metal ions.^[^ ^220^ ^]^ This enhanced binding of single‐stranded nucleic acids is likely due to their greater conformational flexibility and increased exposure of base groups compared to double‐stranded nucleic acids. Another crucial factor affecting the differences in binding behavior is related to the surface areas of each clay.^[^ ^221^ ^]^ Specifically, large double‐stranded DNA molecules exhibited distinctive binding patterns to the clays, with HNT showing higher efficiency in binding large nucleic acids compared to oligonucleotides. The addition of divalent ions further improves the binding of large DNAs, with circular DNAs displaying the strongest affinity. In contrast, kaolinite was much less effective in binding large nucleic acids compared to HNT but shows significant efficiency in binding small single‐stranded RNAs (ssRNAs) in the presence of Mg^2+^ ions. However, few studies have been conducted on kaolinite clay and its interaction with oligonucleotides. Despite increasing interest in the role of mineral clays in biomolecular chemistry, there remains limited research on the interactions between kaolinite and oligonucleotides, leaving their potential relatively underexplored compared to other nanoclays.

**Figure 7:**
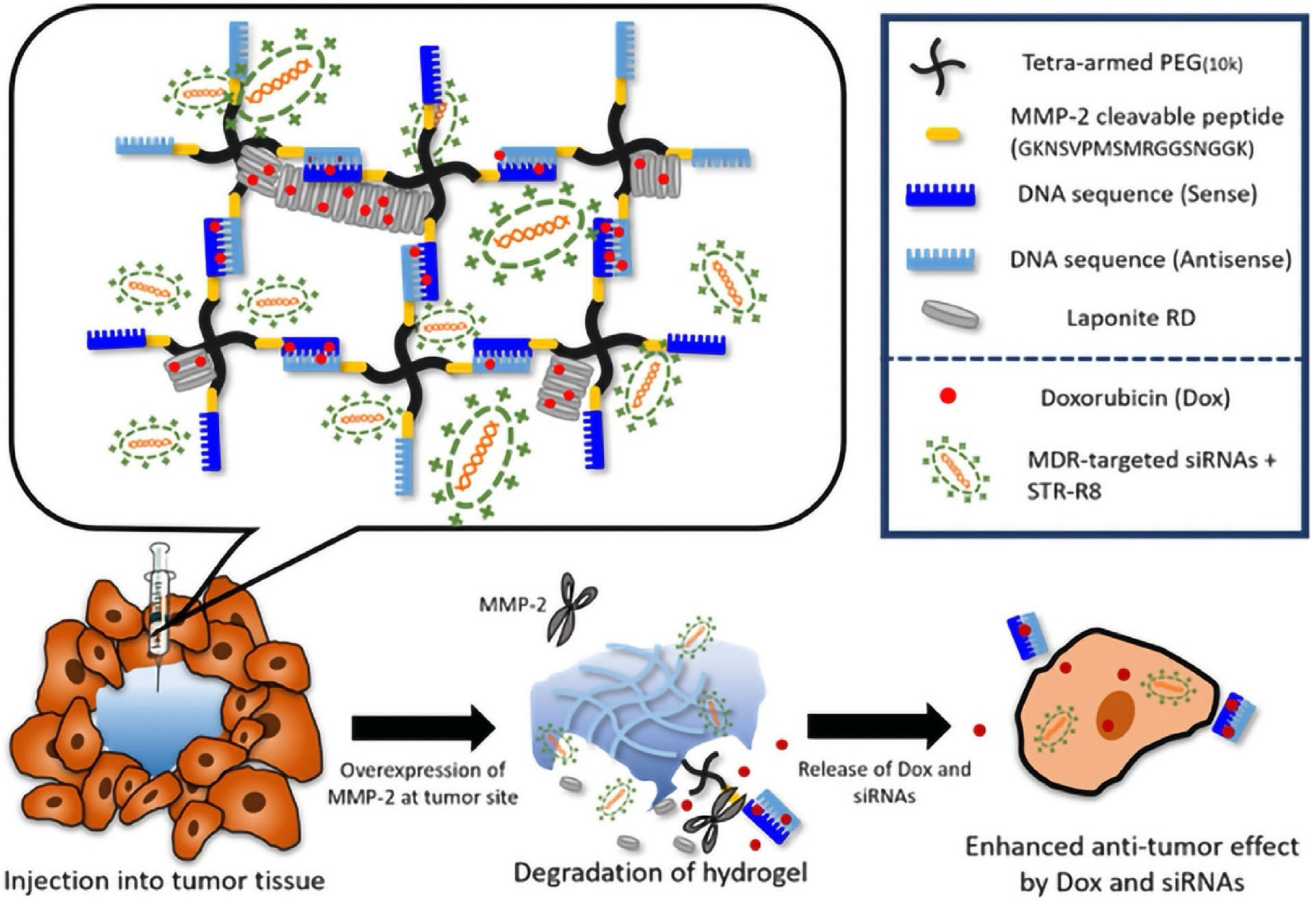
Representation of the DNA‐crosslinked hydrogel network structure and its degradation mechanism. Reproduced with permission.^[^ ^223^ ^]^ Copyright 2023, Elsevier.

A similar observation can be made for laponite nanoclay, a member of the smectite clay family along with montmorillonite. Laponite is disk‐shaped and is able to form a self‐assembling gel through face‐to‐face or face‐to‐edge electrostatic interactions between its nanoplatelets, enabling the intercalation of drug molecules.^[^ ^222^ ^]^ The incorporation of laponite nanoclay enhances the hydrogel's mechanical properties by establishing physical interactions within the hydrogel network. For instance, a study reports the use of laponite nanoclay as a carrier for doxorubicin, employing an intercalation process for drug loading (**Figure** **7**). Additionally, small interfering RNAs were also complexed and encapsulated within laponite hydrogel,^[^ ^223^ ^]^ which can be directly injected into the target site, forming hydrogel in situ for localized delivery with minimal invasiveness, thereby enhancing therapeutic potential.

Nano‐sized acicular materials, such as sepiolite, exhibit strong affinity for nucleic acids and can induce the Yoshida effect.^[^ ^224^ ^]^ Notably, DNA adsorbed onto these materials is protected from nucleolytic degradation mediated by enzyme, maintaining its stability. Other bionanocomposites were effectively prepared by directly assembling ASOs with sepiolite.^[^ ^225^ ^]^ Importantly, the efficiency of their adsorption was proved to be adjusted by introducing polyvalent cations into the reaction medium. Additionally, sepiolite has demonstrated the dual capability of adsorbing and releasing DNA, meeting essential requirements for its use as a carrier in cells.

The interactions between oligonucleotides and sepiolite and montmorillonite were systematically investigated by Kawamura et al.^[^ ^226^ ^]^ The results demonstrated that oligonucleotides bind to montmorillonite in the presence of MgCl_2_, whereas binding to sepiolite occurs both with and without MgCl_2_. The primary factors governing these interactions are electrostatic forces and hydrophobic interactions between the oligonucleotide molecules and the mineral surfaces. This was also proved by a previous study, which observed that oligonucleotide adsorption on montmorillonite is influenced by the type of nucleobases present and the polymer chain length.^[^ ^227^ ^]^ Specifically, the hydrophobicity of the nucleobases and the electrostatic interactions between the phosphate groups of the oligonucleotide and the surface charges of the clay were identified as key factors in binding. Furthermore, the presence of Mg^2+^ ions enhances binding affinity by mediating the interactions between the negatively charged phosphate groups of the oligonucleotides and the negatively charged sites on the clay surface.

MMT belongs to 2:1 clay mineral group, consisting of two tetrahedral sheets of SiO_4_ and one octahedral Al_2_O_3_ sheet. The substitution of Al^3+^ with Mg^2+^ or Fe^2+^ generates a negative surface charge on MMT layers, balanced by interlayer cations. Due to its high cation exchange capacity and swelling properties, MMT can intercalate drug molecules and polymers within its interlayer space, releasing them when replaced by cations in the surrounding medium.

Montmorillonite clay has demonstrated the ability to form complexes with various biomolecules, including nucleic acids. Both organic and inorganic ligands were found to enhance DNA adsorption onto montmorillonite and kaolinite when introduced before the DNA.^[^ ^228^ ^]^ Recent studies have successfully utilized modified montmorillonite as carriers for delivering oligonucleotides into cells, protecting them from nuclease degradation.^[^ ^229^ ^]^ Notably, Kawase et al. performed experiments to evaluate the effectiveness of Na‐MMT as a gene delivery system for plasmid DNA encoding enhanced green fluorescent protein (EGFP).^[^ ^230^ ^]^ In their initial in vitro studies using intestinal epithelial cells (IEC‐6), they observed EGFP expression in cells transfected with the Na‐MMT/DNA complexes. Furthermore, they developed clay/plasmid DNA complexes and administered them orally to mice. EGFP production was detected only in mice that received the MMT/DNA complexes, while no EGFP was observed in mice that received the naked plasmid. These findings support MMT's ability to protect TOs and plasmid DNA from the acidic conditions of the stomach and the degrading enzymes in the intestine, enabling their successful delivery. However, the binding mechanism of single‐ or double‐stranded DNA binds to the large, negatively charged surfaces of montmorillonite particles is believed to be influenced by factors such as pH and the concentration and type of metal cations present.^[^ ^231^ ^]^ As previously reported, the amount of nucleotides adsorbed by MMT is affected by temperature and is significantly increased over a wide pH range (4–10).^[^ ^232^ ^]^ Each nucleotide carries a strong negative charge due to the phosphate groups, and it has been suggested that exchangeable surface cations may act as bridges between the phosphates and the negatively charged tetrahedral silica layers on the clay surface.^[^ ^211^ ^]^ Experimental evidence indicates that all tested nucleic acids primarily adsorb onto the surfaces and external edges of the clay, without intercalating into the clay layers.^[^ ^233^ ^]^

The binding of small interfering RNAs to Na‐MMT and Ca‐MMT was investigated by Gujjari et al.^[^ ^234^ ^]^ Their findings revealed weak association of both Na‐MMT and Ca‐MMT with 25mers and 54mers dsRNAs, showing only a slight increase when Mg^2+^ ions were added. In contrast, ssRNAs of 25mers and 54mers exhibited weak binding to Na‐MMT but interacted strongly with Ca‐MMT. The addition of Mg^2+^ ions significantly enhanced the binding of ssRNAs to Na‐MMT. Further evaluation of MMT‐ssRNA interactions revealed that ssRNAs binding, unlike dsRNAs binding, occurs via a cation‐bridging mechanism facilitated by divalent metal cations. The compatibility of functional nucleic acids with on montmorillonite adsorption depends on whether such binding disrupts critical functional interactions. For instance, the activity of an aptamer or ribozyme relies on its specific mechanism, its interactions with the clay surface, and the presence of dissolved salts and solution conditions. While some self‐cleaving ribozymes exhibited resilience to adsorption, retaining activity with only moderate loss, this cannot be broadly applied, as demonstrated in recent studies involving the malachite green aptamer.^[^ ^235^ ^]^

### 4.2 Functionalized HNTs for Loading and Delivery of TOs

HNT is a 1:1 clay mineral with a tetrahedral silicate sheet and an octahedral aluminum hydroxide sheet. These sheets are rolled into multilayered tubes with an outer diameter of 40–100 nm, an inner lumen diameter of 10–50 nm, and a length ranging from 0.5 to 2.0 mm.^[^ ^236^ ^]^ As reported in a recent article, halloysite possesses a smooth surface with stochastic distribution of positively‐charged patches that affect the interactions between HNTs and oligonucleotides, including DNA and aptamers.^[^ ^237^ ^]^ HNTs are promising materials with potential applications across different fields, including their use as nanoscale containers for encapsulating biologically active molecules,^[^ ^238^ ^]^ supports for catalyst immobilization, controlled drug delivery, bioimplants, and protective coatings (e.g., anticorrosion or antimolding).^[^ ^239^, ^240^ ^]^ The tubular structure of HNTs enables the binding of high‐molecular‐weight drug molecules and polymers, either within the lumen or on the surface, depending on charge differences. The outer surface of HNTs is negatively charged, while the inner lumen surface is positively charged over a wide pH range,^[^ ^241^ ^]^ allowing for selective drug loading. This capacity can be further enhanced by etching the lumen, and the tube openings can be sealed to prevent premature release of the loaded substances, making HNTs a powerful tool for controlled and targeted drug delivery. The concept of using halloysite as a nanocontainer for drug loading and release was initially introduced by Price et al.^[^ ^242^ ^]^ who employed the nanotube as a carrier for oxytetracycline HCl (a water‐soluble antibiotic), khellin (a lipophilic vasodilator), and nicotinamide adenine dinucleotide (NAD) (a key coenzyme). Later, Veerabadran et al. also demonstrated the ability of halloysite to load and release of dexamethasone and furosemide in a controlled manner.^[^ ^243^ ^]^

A thorough investigation into the binding of nucleotides and DNA with HNTs represents a crucial first step toward exploring the potential of this nanoclay for delivery applications. A study by Batasheva et al. showed that the binding affinity of various nucleotides to nanoclay was generally low, with and without MgCl_2._^[^ ^244^ ^]^ However, MgCl_2_ significantly enhanced the binding of larger molecules, such as DNA and polyAU. ζ‐potential measurements confirmed the successful modification of nanotubes with DNA and nucleotide species. Unlike previous methods that combined DNA with halloysite through high‐speed vibration milling, DNA binding to halloysite in solution with MgCl_2_ is a more controlled approach for preparing DNA‐modified HNTs. Additionally, a low‐cost and one‐pot method using a mechanochemical reaction for the preparation of DNA‐coated HNTs has been reported in the literature.^[^ ^245^ ^]^

Clay minerals are commonly used as carriers in biomedicine to protect genetic materials within the body, particularly in the gastrointestinal tract. Among these, HNTs stand out as highly suitable material for gene transfer due to their positively charged lumen and negatively charged outer surface.^[^ ^246^ ^]^ For example, Yang et al. developed chitosan oligosaccharide‐grafted HNTs (HNTs‐g‐COS) as a carrier for doxorubicin (DOX) to treat breast cancer both in vitro and in vivo.^[^ ^247^ ^]^ The resulting DOX@HNTs‐g‐COS demonstrated low hemolysis, good biocompatibility, and controlled drug release in vitro. In cell experiments, DOX@HNTs‐g‐COS effectively induced apoptosis in MCF‐7 breast cancer cells. In vivo studies revealed that DOX@HNTs‐g‐COS exhibited superior tumor inhibition and caused fewer cardiomyocyte ruptures than free DOX. Moreover, the formulation showed no toxicity in vital organs, including heart, lung, kidney, and liver tissues.

Massaro et al. reported multifunctional cationic carriers based on halloysite, designed for both gene therapy and fluorescent detection but also exhibiting antioxidant properties.^[^ ^248^ ^]^ Nitrogen‐doped carbon dots (HNT‐CDs) were covalently bound to the outer surface of halloysite, creating fluorescent HNTs. To explore their potential for oral delivery of nucleic acids, calf thymus DNA (ct‐DNA) was used as a model. The ct‐DNA formed strong electrostatic interactions with HNT‐CDs and was then gradually released under physiological conditions through a dialysis membrane. This controlled release is essential for ensuring the efficient and targeted DNA delivery following oral administration.

Further progress in improving the interaction behavior between HNTs and oligonucleotides has been made by Rawtani et al.^[^ ^249^ ^]^ The tubular structure of halloysite can enable the formation of silver nanoparticles (AgNPs) on its surface, resulting in AgNP–HNT composites. These composites leveraged halloysite as a support matrix for embedding AgNPs, enhancing DNA‐binding capacity and overall functionality. This study demonstrated that AgNP–HNT composites not only extend the shelf life of AgNPs but also offer promising potential for various biological and medical applications.

Gene silencing has recently emerged as a promising therapeutic approach for human diseases, with siRNA identified as one of the most promising silencing mechanisms. However, the major challenge lies in the intracellular delivery of siRNA to specific tissues and organs expressing the target gene. HNTs have shown potential for nucleic acid delivery, addressing issues related to inefficient application, easy degradation, and toxic side effects associated with siRNA. Liu et al. demonstrated that HNT‐based delivery of siRNA improves serum stability and reduces off‐target effects while preserving silencing efficacy.^[^ ^250^ ^]^ The HNTs/siRNA complex not only protects siRNA from the harsh biological environment but also passively targets tumor tissues both in vitro and in vivo, highlighting their potential as cancer cell‐specific drug carriers. Specifically, selective knockdown of the RIPK4 gene using HNTs/siRIPK4 inhibited bladder cancer growth and progression by suppressing proliferation and inducing apoptosis. Generally, in early‐phase clinical studies, liposomes and polymer‐based particles used for systemic delivery of anticancer siRNA tend to accumulate in the liver or be excreted via kidney filtration, limiting their concentration in tumors. In contrast, in vivo delivery of siRIPK4 via HNTs resulted in targeting and accumulation within tumors. This behavior is attributed to the small diameters of siRIPK4 and HNT particles, which enable the complex to traverse the hepatic sinusoidal endothelium and glomerular filtration barrier, facilitating access to these tissues.

HNTs also demonstrate significant potential for brain drug delivery due to their nontoxicity for endothelial cells, ability to release drugs gradually over varied time spans, and attraction to blood–brain barrier cells. Notably, loading halloysite with ionomycin enhanced the calcium response in brain microvascular endothelial cells and significantly prolonged delivery compared to nonencapsulated ionomycin for the treatment of brain cancer cells.^[^ ^251^ ^]^ Saleh et al. demonstrated that HNTs loaded with diazepam or xylazine can permeate the BBB using an intranasal administration.^[^ ^252^ ^]^ Recently, it was proved that HNTs containing rhodamine isothiocyanate and ionomycin can penetrate the rat‐brain microvascular endothelial cells followed by a prolonged 24 h drugs release.^[^ ^253^ ^]^

**Figure 8:**
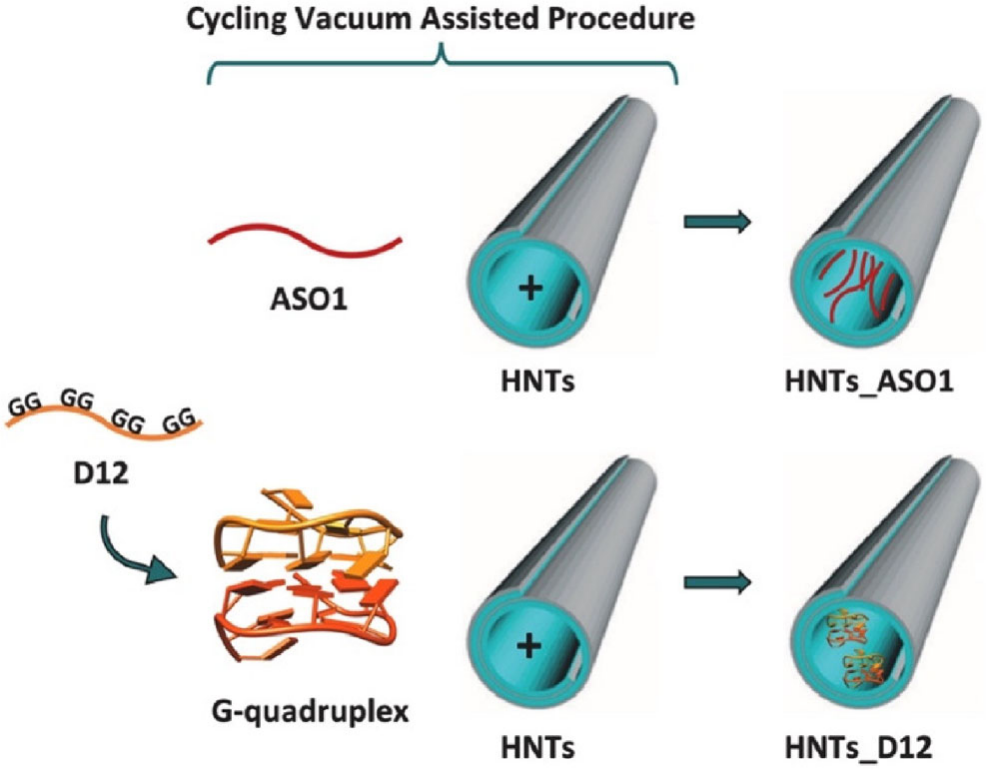
Schematic representation of ASO1 and D12 aptamer loading into the halloysite cavity. ASO1 is a single‐stranded, unstructured oligonucleotide, whereas D12 adopts a dimeric G‐quadruplex structure, where two monomers stack through *π*–*π* interactions. Reproduced with permission.^[^ ^255^ ^]^ Copyright 2025, Elsevier.

A recent study demonstrated the encapsulation of a G‐quadruplex‐forming DNA aptamer (D12) and an antisense oligonucleotide (ASO1), both with potential therapeutic applications for prion diseases,^[^ ^26^, ^254^ ^]^ within the lumen of HNTs using a cyclic vacuum‐assisted protocol in aqueous medium (**Figure** **8**).^[^ ^255^ ^]^ The loading of both D12 and ASO1 into HNTs was achieved through the following steps: 1) addition of 0.05 g of halloysite into a TOs aqueous solution (50 μmol cm^−3^) at pH 7; 2) ultrasonication for 5 min followed by magnetic stirring for 2 h; 3) the resulting stable dispersions were subjected to low‐pressure conditions (*P* = 0.01 atm) for 30 min, followed by 15 min of magnetic stirring, and this cycling vacuum step was repeated three times; 4) separation of the dispersed phase (halloysite loaded with D12 or ASO1) from the solvent by centrifugation at 6000 rpm for 20 min; and 5) rinsing of the hybrid material with water to remove unbound TOs.

Among the two oligonucleotides, ASO1 exhibited higher loading efficiency than D12. Specifically, the amount of ASO1 and D12 loaded into HNTs was reported to be 4.12 and 3.07 μmol g^−1^, respectively. The results indicated that the formation of the hybrid nanomaterial was driven by electrostatic interactions between the negatively charged oligonucleotides and the positively charged inner surface of halloysite, leading to enhanced colloidal stability compared to unmodified clay nanotubes. Accordingly, the zeta potential values of the HNTs_TOs hybrids (−39.8 ± 0.8 and −38.1 ± 0.9 mV for HNTs_ASO1 and HNTs_D12, respectively) were more negative than that of pristine halloysite (−24.7 ± 0.8 mV).

Notably, the encapsulation of negatively charged aptamers within the positive halloysite lumen resulted in a reduction of oligonucleotide release kinetics. This behavior suggests that the therapeutic action of TOs can be extended and modulated over time. In contrast, the intercalation of TOs within other clays, such as montmorillonite, does not produce a comparable reduction in release rate. These findings support the potential of HNTs as nanocarriers for therapeutic applications in neurodegenerative diseases, encouraging further research in this direction.

Recent investigations have deeply explored the use of HNTs as carriers for cancer treatment in in vivo studies. Multifunctional HNT nanocarriers were designed for the delivery of therapeutic antisurvivin siRNA, while also enabling intracellular tracking.^[^ ^256^ ^]^ The goal was to reduce the levels of survival protein as it inhibits apoptosis and promotes proliferation of pancreatic cancer cells (PANC‐1). In these HNT‐siRNA complexes, PEI‐modified HNTs served as the vector, siRNA as the therapeutic agent, and CdSe quantum dots as the tracking agent. The functionalized HNTs (f‐HNT) carriers demonstrated excellent biocompatibility, effective intracellular transport, and high siRNA delivery efficiency. Additionally, siRNA delivered via f‐HNTs successfully induced the knockdown of the target survivin gene in PANC‐1 cells, enhancing its antitumor effects.

Literature reports the synthesis of supramolecular complexes based on HNTs evaluating their cytotoxicity and impact on cellular structures. Specifically, the potential of DNA‐wrapped HNTs (HD) as a promising drug delivery carrier has been investigated, with doxorubicin loaded onto HD as a model anticancer agent.^[^ ^257^ ^]^ The DOX‐loaded, DNA‐wrapped HNTs (HDD) demonstrated a sustained release of DOX over two weeks, without any initial burst, indicating controlled and delayed drug release within cells. Moreover, HDD was shown to alter the cytoskeleton organization of human lung cancer cells.

Another study previously explored the delivery of ASOs using HNTs as nanocarriers. This research investigated a novel gene delivery system based on HNTs for the loading and intracellular delivery of antisense oligodeoxynucleotides (ASODNs). f‐HNTs were employed as carriers, with ASODNs targeting the survivin gene. To enable biofunctionalization, HNTs were initially modified on the external surface with γ‐aminopropyltriethoxysilane. In vitro cytotoxicity studies conducted using the MTT assay revealed a significant increase in cytotoxicity. The results demonstrated that f‐HNT complexes effectively improved intracellular delivery and enhanced the bioactivity of ASODNs via the nanotube carrier, highlighting their potential as novel and promising vectors for gene therapy applications.^[^ ^9^ ^]^

Another approach for siRNA delivery involved the functionalization of HNTs by grafting dendrimers. Kurczewska et al. were the first to report on HNTs functionalized with PAMAM dendrimers, which were developed as vectors for therapeutic compounds.^[^ ^258^ ^]^ The presence of the dendrimer positively influenced the adsorption and/or release of all the studied acidic drugs. Notably, the release of salicylic acid decreased only after the halloysite surface was functionally modified with the PAMAM dendrimer. These findings suggest that functionalizing halloysite surfaces with polyamidoamine dendrimers can significantly enhance the properties of halloysite‐based drug carriers.

Functionalized HNTs are therefore considered as innovative and promising nanovehicles for gene delivery in tumor treatment. Polyethyleneimine (PEI) was grafted onto HNTs to bind plasmid DNA (pDNA) labeled with green fluorescent protein. PEI‐g‐HNTs demonstrated significant transfection efficiency in tumor cells.^[^ ^259^ ^]^ However, the cytotoxicity of these complexes may hinder their broader application in gene therapy. Compared to PEI alone, PEI‐g‐HNTs exhibit lower cytotoxicity, while still being positively charged and able to bind DNA tightly. Notably, PEI‐g‐HNTs/pDNA complexes showed significantly higher transfection efficiency in both 293T and HeLa cells compared to PEI/pDNA complexes, with HeLa cell transfection reaching 44.4%. Additionally, PEI‐g‐HNTs/pDNA complexes exhibited greater protein expression than PEI/pDNA complexes.

**Figure 9:**
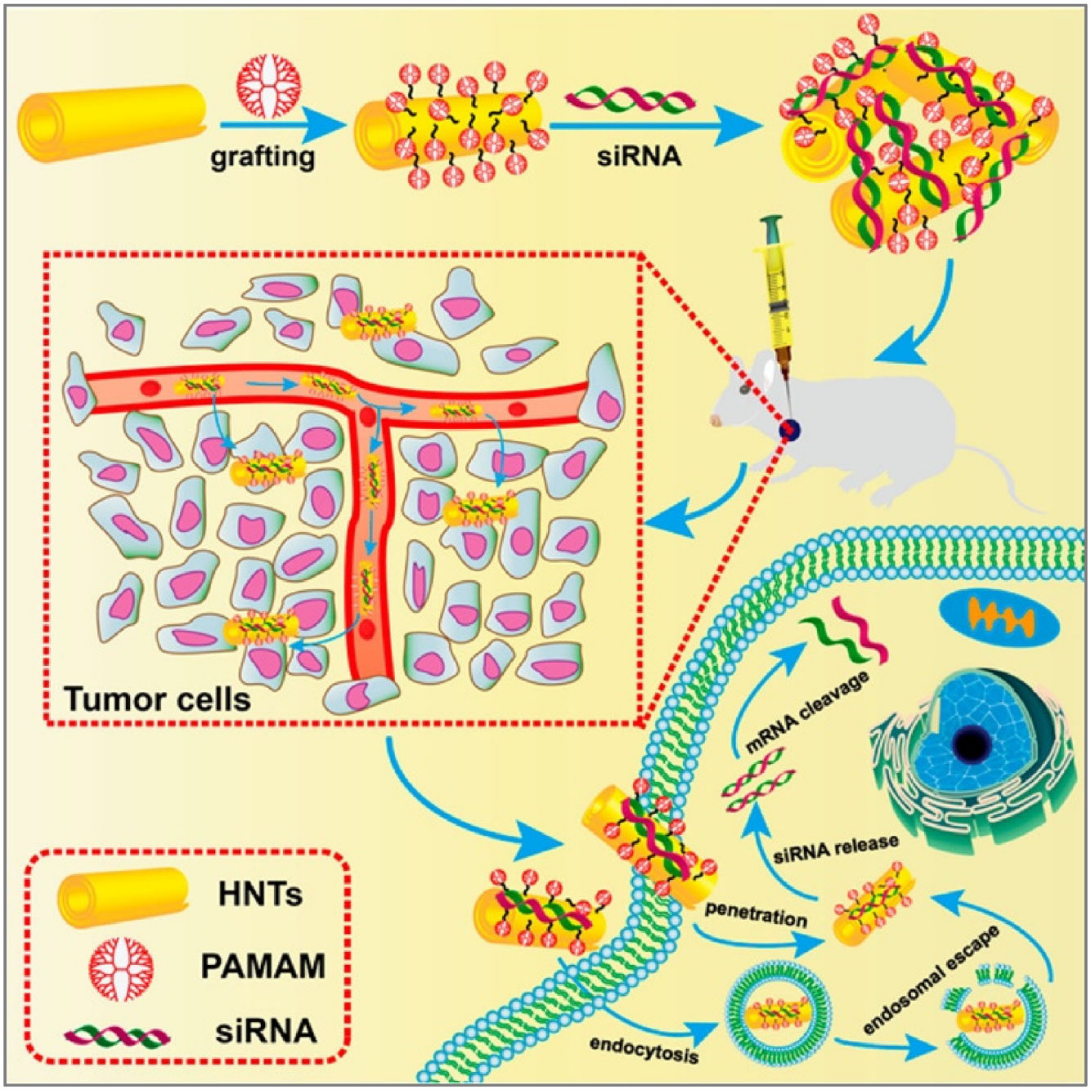
Schematic illustration of PAMAM‐g‐HNTs/siRNA complex and its intracellular process in tumor cells. Reproduced with permission.^[^ ^260^ ^]^ Copyright 2018, American Chemical Society.

In contrast, polyamidoamine‐grafted HNTs (PAMAM‐g‐HNTs) were developed for siRNA loading, with the aim of enhancing intracellular delivery of siRNA for cancer treatment through gene therapy. A schematic representation illustrating the preparation of the PAMAM‐g‐HNTs/siRNA complexes and their intracellular delivery in tumor cells is provided in **Figure** **9**. In vivo studies demonstrated that PAMAM‐g‐HNTs combined with a siRNA for vascular endothelial growth factor mRNA exhibited significant anticancer efficacy in mice, while the nanocarrier system exhibited negligible toxicity to major organs.^[^ ^260^ ^]^ Therefore, PAMAM‐g‐HNTs represent a promising approach as novel nanovectors for siRNA delivery and gene therapy in cancer. However, further research is required to assess the performance of modified HNTs in specific therapeutic applications.

## 5 Summary and Outlook

Aptamer‐based therapeutics have emerged as a promising strategy in the treatment of neurodegenerative diseases due to their remarkable specificity, high affinity, and versatility in targeting pathogenic proteins. In particular, G‐quadruplex‐forming aptamers exhibit unique biophysical properties that enable them to interact with key molecular targets associated with Alzheimer's, Parkinson's, and Huntington's diseases, MS, and prion diseases. Despite their potential, aptamer applications face substantial challenges, including enzymatic degradation, rapid renal clearance, and limited permeability across biological barriers such as the blood‐brain barrier. Recent advancements in nanocarrier systems, such as clay‐based nanomaterials like HNTs and montmorillonite, have provided innovative solutions to improve the stability, bioavailability, and targeted delivery of TOs, including aptamers.

Clay‐based nanocarriers have shown potential in overcoming the BBB for drug delivery, with functionalization or combination with other agents further enhancing their ability to cross it. For example, HNTs can be modified with brain‐targeting ligands, improving their efficiency in delivering therapeutic molecules to the brain. Additionally, the incorporation of divalent metal ions can further enhance their binding and transport capabilities.

Looking ahead, integrating aptamer technology with nanocarriers presents exciting opportunities to overcome current limitations and expanding the therapeutic potential of TOs, especially for neurodegenerative diseases. Future research should focus on optimizing the physicochemical properties of aptamers to enhance their resistance to nucleases while preserving their high binding affinity. Additionally, further investigations are required to refine nanocarrier systems to ensure efficient encapsulation, controlled release, and targeted delivery of aptamers to disease sites. Such advancements would minimize off‐target effects and improve therapeutic outcomes.

The potential of aptamer‐based therapies could be significantly enhanced by leveraging advancements in computational modeling, AI‐assisted aptamer design, and high‐throughput screening techniques. ML approaches may improve the accuracy of aptamer–target interaction predictions, accelerating the discovery of novel therapeutic candidates. Moreover, combining aptamers with other oligonucleotide‐based strategies, such as ASOs and siRNAs, could lead to synergistic effects, enhancing their overall therapeutic efficacy.

The future clinical translation of aptamer‐nanocarrier systems will also require extensive preclinical and clinical studies to evaluate their safety, pharmacokinetics, and biodistribution. Regulatory approval processes must address key concerns regarding immunogenicity, long‐term stability, and large‐scale production feasibility. Despite these challenges, the convergence of nanotechnology and nucleic acid therapeutics holds great promise for developing targeted, efficient, and minimally invasive treatments for neurodegenerative diseases. By addressing these critical issues, aptamer‐based therapeutics have the potential to significantly advance precision medicine and introduce novel therapeutic strategies for otherwise intractable neurodegenerative disorders.

## Conflict of Interest

The authors declare no conflict of interest.

## Author Contributions

**Valentina Arciuolo**: visualization, writing—original draft, writing—review & editing. **Federica D’Aria**: visualization, writing—original draft. **Maria Rita Caruso**: visualization, writing—original draft. **Martina Maria Calvino**: visualization, writing—original draft. **Jussara Amato**: supervision, writing—original draft, writing—review & editing. **Giuseppe Lazzara**: supervision, writing—original draft, writing—review & editing. **Stefana Milioto**: conceptualization, supervision, writing—review & editing. **Concetta Giancola**: conceptualization, supervision, writing—original draft. **Giuseppe Cavallaro**: conceptualization, funding acquisition, writing—original draft, writing—review & editing. **Bruno Pagano**: conceptualization, funding acquisition, supervision, writing—original draft, writing—review & editing.
